# Localized molecular chaperone synthesis maintains neuronal dendrite proteostasis

**DOI:** 10.21203/rs.3.rs-3673702/v1

**Published:** 2023-12-11

**Authors:** Maria Vera Ugalde, Célia Alecki, Javeria Rizwan, Phuong Le, Suleima Jacob-Tomas, Jia Ming Xu, Sandra Minotti, Tad Wu, Heather Durham, Gene Yeo

**Affiliations:** McGill University; McGill University; McGill University; University of California, San Diego; McGill University; McGill University; McGill University; McGill University; McGill University; University of California, San Diego

**Keywords:** Neuron, mRNA, protein homeostasis, dendrite, localized translation, neurodegeneration, heat shock protein, single-molecule fluorescence microscopy, amyotrophic lateral sclerosis

## Abstract

Proteostasis is maintained through regulated protein synthesis and degradation and chaperone-assisted protein folding. However, this is challenging in neuronal projections because of their polarized morphology and constant synaptic proteome remodeling. Using high-resolution fluorescence microscopy, we discovered that neurons localize a subset of chaperone mRNAs to their dendrites and use microtubule-based transport to increase this asymmetric localization following proteotoxic stress. The most abundant dendritic chaperone mRNA encodes a constitutive heat shock protein 70 family member (HSPA8). Proteotoxic stress also enhanced *HSPA8* mRNA translation efficiency in dendrites. Stress-mediated *HSPA8* mRNA localization to the dendrites was impaired by depleting fused in sarcoma—an amyotrophic lateral sclerosis-related protein—in cultured mouse motor neurons and expressing a pathogenic variant of heterogenous nuclear ribonucleoprotein A2/B1 in neurons derived from human induced pluripotent stem cells. These results reveal a crucial and unexpected neuronal stress response in which RNA-binding proteins increase the dendritic localization of *HSPA8* mRNA to maintain proteostasis and prevent neurodegeneration.

## Introduction

Cells have developed intricate mechanisms to maintain proteostasis—that is, to ensure that proteins are synthesized, folded, and degraded as needed. This is particularly challenging for neurons, because their complex polarized morphology includes projections that can span long distances and require constant proteome adjustments to respond to neuronal stimuli ^1–7^. Neuronal activity remodels the axonal terminal proteome ^8–10^. Likewise, stimulating individual dendritic spines triggers unique proteome changes independently from other spines ^11–14^. Neurons remodel local proteomes through the targeted distribution and regulation of the protein synthesis and degradation machinery (*e.g*., the proteasome and autophagy system to degrade damaged and unnecessary proteins) ^15–20^. Neurons ensure timely and efficient protein synthesis through an at-a-distance expression mechanism that relies on localizing specific mRNAs and regulating their stability and translation ^12,21–26^. Thus, neurons tightly regulate the distribution of proteins enriched in axons, dendrites, and synapses by localizing their mRNAs and necessary translation factors to these regions while retaining other mRNAs in the soma ^24,27–30^. Axon- and dendrite-targeted mRNAs contain specific sequence/structure motifs zip codes) recognized by particular RNA binding proteins (RBPs) ^31,32^. Selective interactions between RBPs and motors form unique complexes or neuronal granules ^33–38^, which move mRNAs in both directions by anchoring them to microtubule motors (dynein and kinesins) or membranous organelles for active transport to axons and dendrites ^24,27,39,40^. Some RBPs also prevent mRNA translation during transport and derepress translation in response to local synaptic stimuli ^41–43^.

Successful protein synthesis and targeted degradation require the chaperoning function of heat shock proteins (HSPs). HSPs facilitate the folding of newly synthesized polypeptides into their functional three-dimensional conformations, and subsequently sequester or refold proteins that take on abnormal conformations, preventing aggregation and aberrant interactions in the crowded intracellular environment ^44–47^. Multiple HSPs load onto a misfolded substrate and perform several refolding cycles to restore proper conformation and sustain proteostasis ^48^. HSPs are grouped into families based on their molecular weights ^45,49^. The HSP60, HSP70, and HSP90 families actively promote protein folding in all cell types ^50–52^, and their functions are modulated by diverse co-chaperones ^53–56^. They also cooperate with small HSPs (sHSPs) ^52,53,57^ and HSP110 ^58^ to prevent and resolve misfolded protein aggregation and target misfolded proteins for degradation ^59–62^. Accordingly, subsets of them localize prominently to the dendrites and axons in diverse neuronal types ^63–66^. Intriguingly, neurons subjected to proteotoxic stress have elevated levels of HSP70 and DNAJ (HSP40) family members in their dendrites and synapses ^63,67–69.^

The mechanism underlying HSP subcellular distribution in neurons represents a major knowledge gap. Most studies on induced HSP expression have investigated their upregulation by the transcription factor heat shock factor 1 (HSF1) ^47,70–73^. Recently, mRNAs encoding HSPA8 and HSP90AA were identified in the dendritic transcriptome under basal conditions ^74^. Here, we report that neurons increase the transport and local translation of a subset of HSP mRNAs in the dendrites in response to proteotoxic stress. Combining high-resolution fluorescence microscopy and molecular biology, we characterized changes in the subcellular localization of HSP mRNAs in primary hippocampal and motor neurons subjected to different proteotoxic insults. Fused in sarcoma (FUS) and heterogenous nuclear ribonucleoprotein A2/B1 (HNRNPA2B1), both implicated in amyotrophic lateral sclerosis (ALS), were identified as important regulators of the subcellular distribution of the constitutive HSP70 mRNA, HSPA8, and their actions were essential for dendritic proteostasis during stress.

## Results

### Hippocampal neurons alter HSP mRNA distributions upon stress

**Figure 1:**
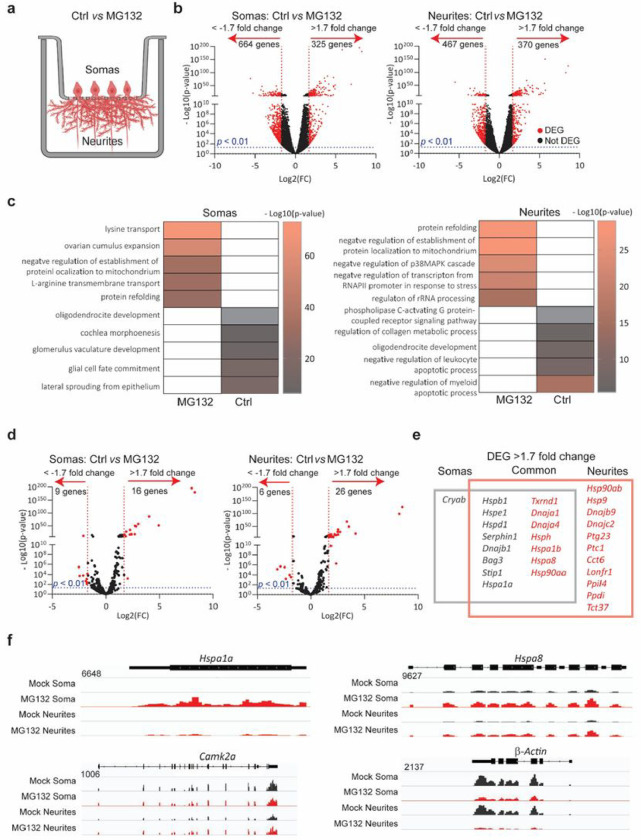
Specific mRNAs are preferentially enriched in the soma or projections of hippocampal neurons after proteostatic stress **(a)** Schematic of primary mouse hippocampal neurons cultured in Transwell membranes to physically separate the soma and neurites for RNA extraction. Neurons were exposed to MG132 or DMSO (Ctrl). **(b)**Volcano plot of differentially expressed genes (DEGs) in the soma or neurites (*n* = 3). Genes up- or down-regulated by > 1.7 fold after MG132 treatment and *P*-values < 0.01 are indicated in red. **(c)**Gene ontology enrichment analysis. Gene ontology categories of the top five biological processes enriched in DEGs in the somas and neurites after MG132 exposure. The color of the bands denotes the extent of upregulation. **(d)**Volcano plot of known chaperone-related genes. Genes up- or down-regulated by > 1.7 fold after MG132 treatment and *P*-values < 0.01 are indicated in red. **(e)**Venn diagram listing the differentially enriched molecular chaperone-related genes in the somas (gray square) and neurites (red square). **(f)** RNA-seq distributions of the *Hspa1a1, Hspa8, Camk2a*, and b-actin loci in the soma and neurites of control and MG132-exposed neurons.

To study how neurons tailor HSP expression to proteostatic demands, we subjected cultures of primary mouse hippocampal neurons to proteotoxic stress by inhibiting the proteasome. These cultures faithfully recapitulate the regulation of mRNA localization and translation in response to neuronal stimuli and the activation of HSP transcription upon stress ^12,75–78^. Dissociated cultures of hippocampi from postnatal day 0 mouse pups differentiate to express features of their mature *in situ* counterparts by day 17. Treatment with the proteasome inhibitor MG132 results in the accumulation of misfolded proteins and protein quality failure—prominent hallmarks of neurodegenerative disorders ^79^. To study MG132-induced changes in subcellular mRNA localization, we isolated the total RNA from somas and projections separately harvested from neurons cultured in Transwell membrane filter inserts ^80^ (Fig. 1a). RNA sequencing (RNA-Seq) and differential expression analysis (DESeq2) revealed previously described transcript signatures specific to somas and projections in steady-state (control (Ctrl)) neurons, *e.g*., dendritic localization of calcium/calmodulin-dependent protein kinase II alpha (CAMKII) and β-actin) ^74,81^. Exposure to MG132 (10 μM for 7 h) significantly changed the expression of hundreds of RNAs between the soma and dendrites. In fact, in gene ontology analyses of the changed transcripts, the only biological function enriched in both compartments was “protein refolding” (Fig. 1c, S1a–S1c **and Table S1).**

Mammals contain over 400 genes encoding molecular chaperones and co-chaperones ^82^; of these, only 16 were upregulated in both fractions with increased enrichment in either the soma (*e.g*., the inducible HSP70 HSPA1A1) or the projections (*e.g*., the constitutive HSP70 HSPA8). Interestingly, while mRNAs for 11 chaperones were specifically increased in neuronal projections, only the mRNA encoding the sHSP CRYAB was enriched in the soma (Fig. 1d–1f). Importantly, co-chaperones colocalized with their chaperone partners and HSP mRNA distributions matched the subcellular locations of their known folding clients. For instance, HSP90s and their co-chaperone P23 were enriched in projections upon stress. In dendrites, HSP90 supports the delivery of α-amino-3-hydroxy-5-methyl4-isoxazolepropionic acid receptors to the spine membrane, which is critical for synaptic transmission in the hippocampus ^83^. Likewise, DNAJs localized with their refolding partners, constitutive (HSPA8) and inducible (HSPA1A) HSP70s. In contrast with the significant upregulation of HSP mRNAs in projections, the levels of CAMKII and β-actin mRNAs, which are well-known to localize to dendrites, were unchanged ^74,81^ (Fig. 1f). These data suggest that neurons identify the need for HSP and co-chaperone mRNAs and distribute them to the same compartments as their client proteins.

### A subset of HSP mRNAs specifically localize to the dendrites upon stress

**Figure 2:**
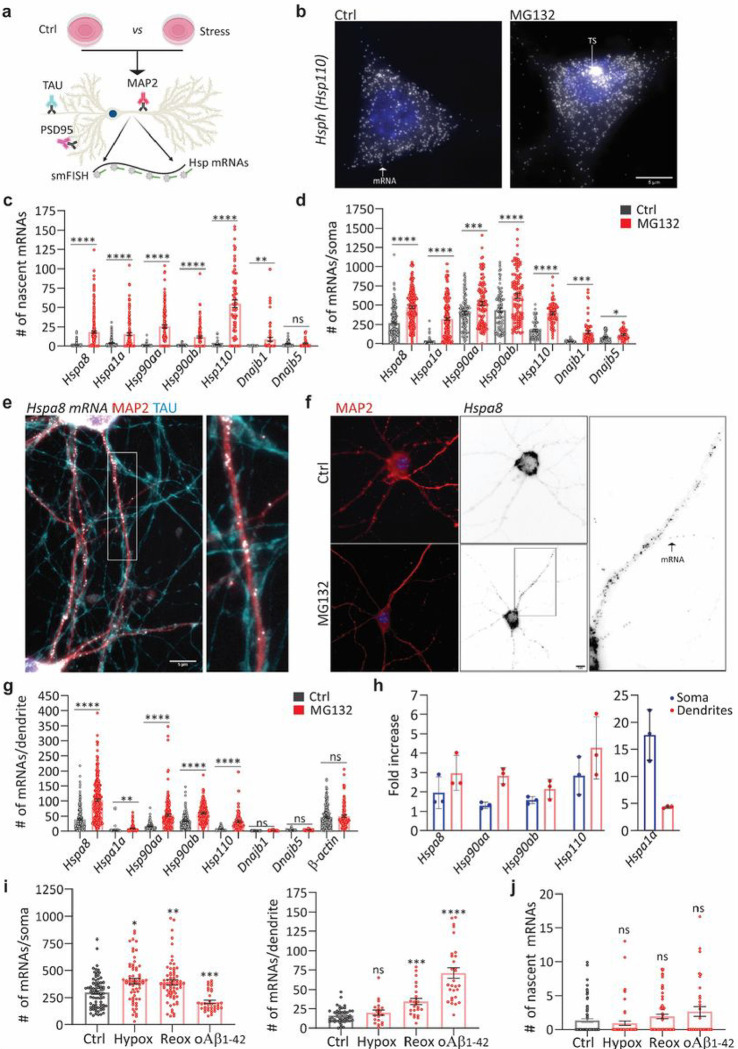
Subcellular distributions of HSP mRNAs in hippocampal neurons upon stress **(a)** Schematic of the combined immunofluorescence (IF) and single-molecule fluorescence *in situ* hybridization (smFISH) protocol used on fixed primary hippocampal neurons. (**b**) smFISH detection of *Hsp110* mRNAs in the soma and nucleus (blue) of control (Ctrl) and MG132-stressed neurons. Arrows in the Ctrl and MG132 images indicate a single mRNA and a transcription site (TS), respectively. (**c, d)** Quantification of nascent transcripts (**C**) and somatic (**D**) HSP mRNAs in Ctrl and MG132-stressed neurons. Data are the mean ± standard error of the mean (SEM) of three independent experiments (*n* = 45–180 neurons total; dots indicate individual values).**(e)** Localization of *Hspa8* mRNA (smFISH, white) in the dendrites (IF: MAP2, red) and axons (IF: TAU, blue) of hippocampal neurons. Scale bar = 5 mm. The square depicts the magnified region. **(f)** Detection of *Hspa8* mRNA (smFISH, black) in dendrites (IF: MAP2, red) in Ctrl and MG132 stressed neurons. The square depicts the magnified region. **(g)** Quantification of dendritic HSP mRNAs in the Ctrl and MG132-stressed neurons in C, D. (**h)** Fold enrichment of HSP mRNAs in the soma and dendrites of MG132-stressed (MG) and Ctrl neurons from the quantifications in C, D, and G. (**i**) Quantification of somatic and dendritic *Hspa8* mRNAs in Ctrl hippocampal neurons and those stressed by hypoxia (Hypox), hypoxia followed by reoxygenation (Reox), or incubation with amyloid beta (1–42) oligomers (oAb_1_-42). (**j**) Quantification of nascent *Hspa8* mRNA in one replicate of (I). Data are the mean ± SEM of two independent experiments (*n* = 25–54 neurons; dots indicate individual values). ****, *P* < 0.0001; ***, *P* < 0.001; **, *P* < 0.01; *, *P* < 0.05; ns, not significant (by unpaired *t*-test).

To define the principles of selective neuronal HSP mRNA localization and to identify proteotoxicity-induced changes in their subcellular distributions, we combined single-molecule fluorescence *in situ* hybridization (smFISH) with immunofluorescence (IF) to localize single mRNAs in primary hippocampal neurons using established markers of dendrites (microtubule-associated protein 2 (MAP2)), axons (microtubule-associated protein tau (TAU)), and spines (postsynaptic density 95 (PSD95); Fig. 2a) ^84,85^. Single mRNAs were identified and quantified using FISH-quant ^86,87^. To collect statistics on mRNA localization across the neuronal morphology, we updated the computational pipeline Analysis of RNA Localization In Neurons (ARLIN ) ^88^. This pipeline was used to validate the RNA-seq data by studying the main HSPs implicated in proteostasis loss during neurodegeneration: HSPA1A, HSPA8, HSP90AA, HSP90AB, and HSP110 ^50,58,66,89,90^. We first analyzed changes in the soma and quantified the significant transcriptional induction and increased concentrations of these constitutive and inducible HSP mRNAs, while the induction of DNAJB1 and DNAJB5 (used as controls) was lower; the same pattern was detected by RNA-seq (Fig. 2b–d).

We next ascribed the mRNAs enriched in neuronal projections to either the axons or dendrites by combining smFISH with IF of TAU and MAP2. Remarkably, we did not detect any HSP mRNAs in the axons of Ctrl or MG132-stressed neurons (Fig. 2e). Instead, the mRNAs of all HSP of interest localized to the dendrites and were significantly enriched by MG132 exposure (Fig. 2f and 2g). However, their concentrations varied greatly; from an average of 100 *Hspa8* mRNAs to only five *Hspa1a* mRNAs per dendrite. *Hspa1a* mRNA was retained in the soma, whereas *Hspa8*, *Hsp90aa*, and *Hsp110* mRNAs were enriched in the dendrites after MG132 stress, confirming the RNA-seq data (Fig. 2h). These results, together with the unchanging distribution of *CamkII* mRNA in the dendrites in response to MG132, strongly suggest that neurons selectively target specific HSP mRNAs to the dendrites upon stress (Fig. 2g and 2h).

Since *Hspa8* was the most abundant dendritic HSP mRNA measured, we verified that its dendritic localization occurs under disease-related stress conditions (Fig. 2i and 2j). We used hypoxia and hypoxia-reoxygenation injury 1% O_2_ for 3 h and 4 h reoxygenation, which generates reactive oxygen species resulting in protein misfolding ^91^ and brain damage ^92,93^ or neuronal exposure to 500 nM of oligomeric amyloid-β peptides for 24 h (oAβ_1–42_) ^94,95^, which accumulate in the hippocampus in Alzheimer’s disease and cause dendritic attrition ^94,95^. Dendritic *Hspa8* mRNA localization significantly increased after reoxygenation or oAβ_1–42_ exposure (Fig. 2e). Notably, although the level of transcriptional induction was much lower than after MG132 exposure, these stresses relocated somatic *Hspa8* mRNAs to the dendrites (Fig. 2j). Therefore, the subcellular localization of *Hspa8* mRNAs to the dendrites is promoted by diverse proteotoxic stresses. These results suggest a common stress-induced regulatory mechanism that directs specific HSP mRNAs to the dendrites.

### Stress promotes HSP mRNA localization to the proximal dendrites and spines

**Figure 3:**
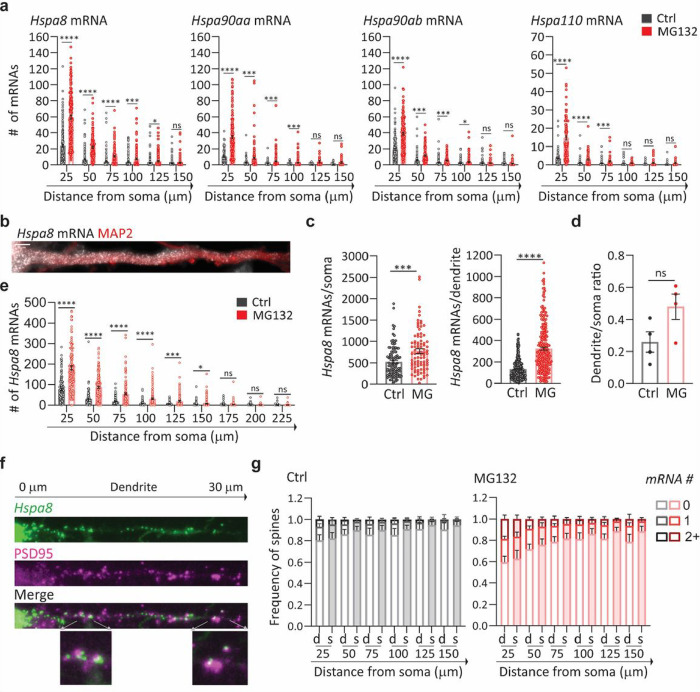
Stress-induced changes in dendritic HSP mRNA localization in primary neurons **(a)** Quantification of the dendritic mRNAs located in 25-mm bins based on their distance from the soma. Data are the mean ± SEM of three independent experiments (*n* = 116–185 dendrites; dots indicate individual dendrites). **(b)** smFISH detection of *Hspa8*mRNAs in the dendrites of an MG132-stressed primary mouse motor neuron stained with MAP2. Scale bar = 5 mm.**(c)** Quantification of somatic and dendritic *Hspa8* mRNAs in Ctrl and MG132-stressed motor neurons. Data are the mean ± SEM of four independent experiments (*n* = 87–100 neurons; dots indicate individual soma and dendrite values). (**d**) Ratio of *Hspa8* mRNA per area of dendrite or soma (in pixels) in Ctrl and MG132-stressed motor neurons analyzed in C. **(e)** Quantification of *Hspa8* mRNAs per 25-mm bin in experiment **c. (f)** Detection of *Hspa8* mRNAs in relation to the dendritic spines, identified by anti-PSD95 IF. The distances shown are in relation to the soma. The lower images show magnifications of mRNAs localizing to the dendritic spines in the areas indicated by the arrows. **(g)** Frequency of dendrites with 0, 1, and 2 or more *Hspa8* mRNAs localizing within 600 nm of the center of the PSD95 IF signal in Ctrl and MG132-stressed primary hippocampal neurons from panel a. Dendritic spines were assigned to 25-mm bins based on their distance from the soma. Six experiments were analyzed. Simulated data are the average of 100 random simulations of the positions of each detected Hspa8 mRNA in the specific dendritic bin. Experimental and simulated data are denoted with d and s, respectively. ****, *P* < 0.0001; ***, *P* < 0.001; **, *P* < 0.01; *, *P* < 0.05; ns, not significant (by unpaired multiple Welch’s *t*-test).

We next investigated whether stress induces changes in the distribution of HSP mRNAs within the dendritic compartment, and particularly in the dendritic spines, which receive synaptic inputs. First, we considered that stress might drive HSP mRNAs into distal dendrites, broadening their distribution. To test this hypothesis, we measured the distance that each mRNA traveled from the soma and grouped them into bins of 25 μm to analyze their dendritic distributions (Fig. 3a). Stress induced significant increases in the number of *Hspa8, Hsp90aa, Hspa90ab*, and *Hsp110* mRNAs in the bins proximal to the soma. However, no significant increases in any of the mRNAs were observed at distances ≥ 100 μm from the soma. Thus, although the dendritic concentration of HSP mRNAs increased upon MG132-stress, their distribution over the dendritic shaft was comparable to that in non-stressed neurons, with more mRNAs in the proximal dendrites (Fig. 3a).

We examined whether mouse primary motor neurons, which have more extensive and thicker dendrites than hippocampal neurons, similarly regulate *Hspa8* mRNA. Dissociated cultures of embryonic day 13 mouse spinal cords were matured for at least 3 weeks, when cultured motor neurons express properties of their *in situ* counterparts ^96^. Upon MG132 exposure, cultured motor neurons *Hspa8* mRNA concentrations in the soma and dendrites, promoting dendritic localization (Fig. 3b–3d); however, *Hspa8* mRNA was more concentrated in the proximal dendrites in both stressed and non-stressed neurons (Fig. 3e). These results indicate that the regulation of HSP mRNA localization is shared by different neuron types and that their movement towards distal dendrites is constrained or subjected to retrograde transport.

Considering the concentrated localized translation that occurs in the dendritic spines, we hypothesized that they would require relatively more HSPs to prevent aberrant interactions among unfolded proteins. Thus, we used ARLIN to analyzed stress-induced changes in the contiguity between HSP mRNAs (detected by smFISH) and the postsynaptic density (detected by PSD95 IF) ^88^ (Fig. 3f). Quantifying mRNAs within 600 nm of the center of the PSD95 signal showed that MG132 stress increased the number of spines containing at least one *Hspa8, Hsp90aa, Hsp90ab*, or *Hsp110* mRNA (Fig. 3f and 3g **and Fig. S2a**). We used ARLIN to bin the dendritic shafts into 25 μm segments and organized them by distance from the soma to examine changes in the number of HSP mRNAs. The frequency of spines containing HSP mRNAs was higher in the proximal dendrites than in the distal, with up to ~ 20% and 40% of spines localizing *Hspa8* mRNAs in control and MG132-stressed neurons, respectively (Fig. 3g). To determine whether localization near a dendritic spine was regulated or due to increased HSP mRNA density, we used the ARLIN segmentation module to run random simulations of HSP mRNA localizations while maintaining the position of the PSD95 signal. The contingency between all HSP mRNAs and dendritic spines was similar to that in random simulations over the dendritic shafts of control and MG132-stressed neurons, with no significant differences (*P* > 0.05 (unpaired *t*-test)) observed between the experimental and simulated data in each bin (Fig. 3i **and Fig. S2a**). Given the density of the cultures, we were unable to quantify mRNAs in the distal dendrites (> 150 μm from the soma) without introducing errors in their assignment to specific dendrites. We conclude that the increased localization of HSP mRNAs in dendrites upon stress increases the number of spines containing them.

### Active transport of individual HSP mRNAs in dendrites

**Figure 4:**
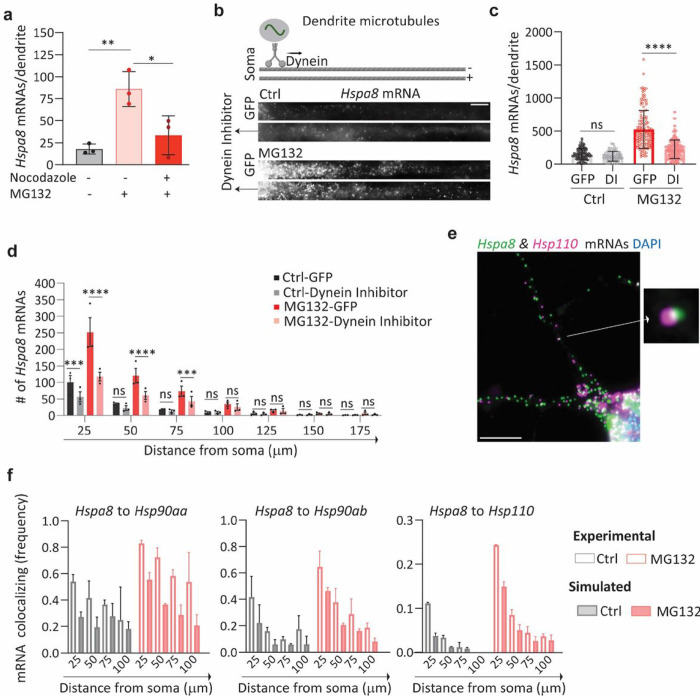
Individual HSP mRNAs are actively transported to the dendrites **(a)** Quantification of dendritic HSPA8 mRNAs in Ctrl neurons and MG132-stressed neurons with and without nocodazole exposure. Data are the median ± SD of three independent experiments. **(b)** Schematic of microtubule orientation and dynein transport in dendrites. smFISH detection of *Hspa8* mRNAs in the dendrites of Ctrl and MG132-stressed neurons. Scale bar = 5 mm. **(c)** Quantification of somatic and dendritic *Hspa8* mRNAs in Ctrl and MG132-stressed motor neurons microinjected with a plasmid expressing GFP or a Dynein Inhibitor (DI). Data are the mean ± SEM of three independent experiments (n = 102–248 dendrites). **(d)** Quantification of *Hspa8* mRNAs per 25-mm bin in experiment **c**. **(e)** Two-color smFISH detection of *Hspa8* and *Hsp110* mRNAs in a primary hippocampal neuron stressed with MG132. The square shows a magnified view of two colocalization mRNAs. **(f)** Frequency of colocalization (< 700 nm away) between *Hspa8* mRNA and *Hsp90aa*, *Hsp90ab*, or *Hsp110* mRNAs in each 25-mm dendrite bin of Ctrl and MG132-stressed primary hippocampal neurons. Simulated data are the average of 100 random simulations of the positions of each detected *Hspa90aa*, *Hspa90ab*, or *Hsp110* mRNA in a specific dendritic bin. Two independent experiments were performed (*n* = 100–200 dendrites total).

We envisioned two non-exclusive mechanisms to promote dendritic *Hspa8* mRNA localization upon stress: active mRNA transport from the soma to the dendrites or enhanced mRNA stability in the dendrites. To distinguish them, we disrupted microtubule polymerization with nocodazole to prevent intracellular transport. The significant reduction in the number of dendritic *Hspa8* mRNAs upon combined MG132/nocodazole treatment confirmed the importance of active transport from the soma to increase dendritic *Hspa8* mRNA levels upon stress (Fig. 4a). As such, longer exposure to MG132 favored dendritic increases in HSPA8 mRNA over a somatic increase, and this subcellular distribution was maintained after MG132 was withdrawn (**Fig. S3a and S3b**). Thus, stress triggered an initial transport of *Hspa8* mRNAs to dendrites that remained stable during recovery, suggesting RBP-mediated transport.

mRNAs are transported in dendrites bidirectionally by motor proteins: dynein for minus-end and kinesin for plus-end movement along microtubules. To identify the motor protein directing *Hspa8* mRNAs to dendrites, we considered they harbor microtubules oriented in both plus and minus directions, while axons feature only plus-end-out oriented microtubules (Fig. 4b)^97^. Since dynein is the only motor exiting the soma using dendrite-specific (minus-end out) microtubules, we propose it directs HSP mRNAs to dendrites. To test this hypothesis, motor neurons were microinjected in the nucleus with a plasmid expressing a dominant negative dynein inhibitor CC1 fused to GFP (a gift of Dr. Adam Hendricks) ^98^ or GFP alone. CC1 blocks the interaction between Dynein and Dinectin important for dynein-mediated cargo movement in cells. Neurons expressing CC1 had a significant decrease in dendritic *Hspa8* mRNA levels upon MG132 exposure (Figs. 4b and 4c). Under permissive conditions, *Hspa8* mRNA dendritic decreased by CC1 expression was only significant in the most proximal 25 μm of dendrite (Fig. 4d). This result suggests a dynein-dependent targeting of *Hspa8* mRNA to dendrites upon stress.

RNAs, proteins, and components of the translation machinery phase separate into neuronal granules of ~ 700 nm in diameter with distinctive compositions ^34,37,99^. The concomitant localization and similar dendritic distribution of all HSP mRNAs suggested that they can be assembled and transported in the same neuronal granules. To identify mRNAs traveling with the most abundant dendritic mRNA, *Hspa8*, we performed two-color smFISH in hippocampal neurons to detect *Hsp90aa, Hsp90ab*, or *Hsp110* localizing within 700 nm of, and thus coexisting with, each *Hspa8* molecule (Fig. 4b). We observed higher frequencies of *Hspa8* coexisted with any of the other HSP mRNAs in MG132-stressed neurons than in non-stressed neurons, and in proximal dendrites than in distal dendrites (Fig. 4c). To differentiate regulated and random mRNA colocalization, we created a new module in ARLIN ^88^ that randomly simulated the positions of *Hsp90aa, Hsp90ab*, or *Hsp110* mRNAs over the dendritic shaft. After binning the shaft into 25 μm segments, ARLIN averaged the shortest distances between *Hspa8* and the closest HSP obtained in a hundred100 random simulations. Although the coexistence between HSP mRNAs significantly increased upon MG132 exposure, the levels were similar between the experimental and simulated data in control and MG132-stressed conditions (Fig. 4c). Similar results were obtained when quantifying the coexistence of *Hsp90aa, Hsp90ab*, or *Hsp110* mRNAs with Hspa8 mRNA (**Fig. S3c**). These results suggest that HSP mRNAs are packaged and transported in individual neuronal granules, as previously reported for other dendritic mRNAs ^100^.

### HSPs concentrate in the same neuronal compartments as their mRNAsvialocalized translation

**Figure 5:**
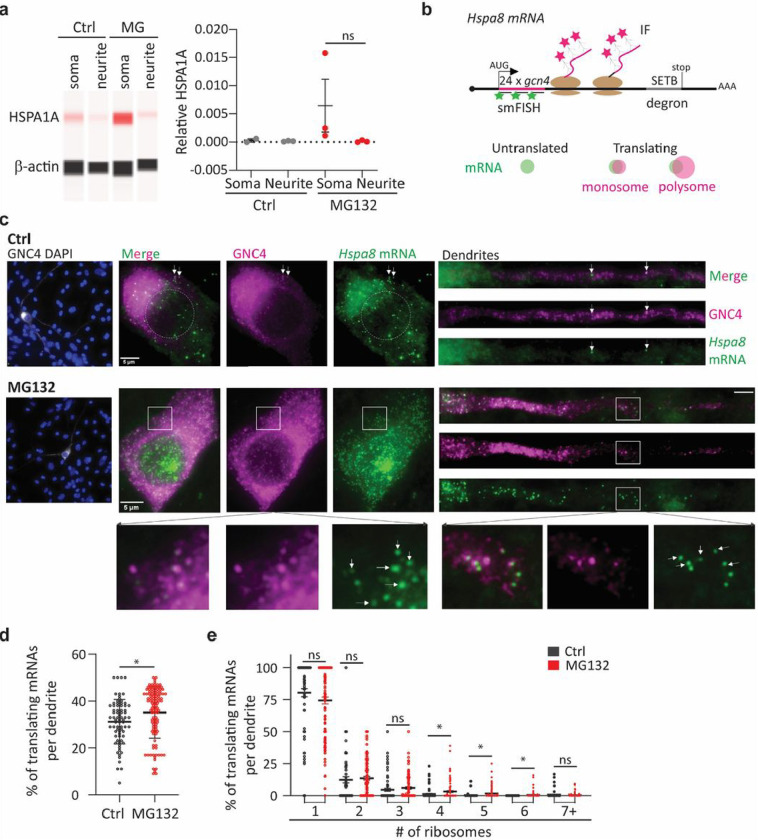
Localized HSP mRNA translation in primary neurons **(a)** Simple Western detection of HSPA1A and b-actin in protein extracts of the somas and neurites of Ctrl and MG132-stressed primary hippocampal neurons. The plot shows the quantification of *Hspa1a*relative to *b-actin*in three independent experiments. **(b)** Schematic of the *Hspa8* single-molecule translational reporter and the IF-smFISH signals expected for untranslated mRNAs and those being translated by a monosome or polysome. Distention among them was based on the intensity of the IF signal colocalizing with the mRNA, which is proportional to the number of nascent peptides produced from an mRNA. **(c)** Representative IF-smFISH images of Ctrl and MG132-stressed primary motor neurons expressing the *Hspa8* single-molecule translational reporter. White arrows indicate translating mRNAs. Squares depict the magnified regions. **(d)** Quantification of the percentage of translated mRNAs per dendrite. Data are the mean ± SD of five independent experiments (*n* = 106–126 dendrites; dots indicate individual dendrites). **(e)** Quantification of nascent peptides colocalizing with translating mRNAs. Dots represent the percentage of mRNAs in the individual dendrites in D being translated by the indicated number of ribosomes. ***, *P* < 0.001; **, *P* < 0.01; *, *P* < 0.05; ns, not significant (by unpaired *t*-test).

Transcript shuttling and local translation is the most efficient way for neurons to target proteins to the dendrites and their spines ^21,22^. We tested whether this regulatory mechanism supports the subcellular distribution of the inducible and constitutive HSP70s (Fig. 5). To quantify the increases in inducible HSP70 (HSPA1A) in the somas and dendrites upon MG132 stress, we cultured hippocampal neurons in Transwell membrane filter inserts ^80^. Proteins were isolated from each compartment separately and analyzed by Simple Western. Exposure to MG132 for 7 h only increased HSPA1A in the soma compartment (Fig. 5a), matching the somatic retention of its mRNA (Fig. 1e and 1h). The mRNA level of the constitutive HSP HSPA8 increased in both compartments and matched the increased HSPA8 IF signal observed in MG132-stressed hippocampal neurons (**Fig. S4a**). These results suggest that localized synthesis determines an HSP’s subcellular distribution.

To investigate whether the mRNAs in each compartment are translated there, we generated a single *Hspa8* mRNA translation reporter using the SunTag translation reporter system ^101–104^ (Fig. 5b). This plasmid reporter contains *Hspa8*’s 5′ and 3′ untranslated regions (UTRs), coding sequence (CDS), and promoter sequences. We placed 24×GCN4 epitopes at the 5′ end of the CDS to detect nascent peptides as soon as they exit the ribosome tunnel. Translating mRNAs are indicated by colocalization of the smFISH and IF signals detecting the GCN4 nucleotide and amino acid sequences, respectively, while individual signals indicate untranslated mRNAs (green) or fully synthesized proteins that have diffused away from their mRNA (magenta; Fig. 5b). We used the IF signal intensity to quantify the number of peptides being synthesized per mRNA as a read out of the number of ribosomes reading an mRNA. The HSPA8 translation reporter resulted in the expression of the expected 125 kDa protein (**Fig. S4b**) and enabled the detection and quantification of single mRNA translation in individual mouse embryonic fibroblasts (MEFs; **Fig. S4c and S4d**). Stressing MEFs with MG132 significantly increased the number of translated mRNAs by an average of 20–45%. To investigate HSPA8 translation in neurons, this plasmid was intranuclearly microinjected into mature cultured motor neurons derived from dissociated murine spinal cords. Like endogenous *Hspa8* mRNA, the dendritic localization of GCN4-HSPA8 mRNA increased upon MG132 exposure (**Fig. S4e**); however, HSPA8 accumulation in the dendrites resulted in a high fluorescent background that obscured the visualization and quantification of individual translation events (**Fig. S4f**). Thus, we added a C-terminal SET binding protein 1 (SETB) degron to decrease the reporter’s half-life and reduce the background under control conditions (Fig. 5b).

Motor neurons were microinjected with the optimized reporter plasmid, cultures were treated with dimethyl sulfoxide (DMSO; Ctrl) or MG132 11 h later, and translation was analyzed by smFISH-IF 7 h later (18 h after microinjection). The short window between injection and detection was critical to quantify the efficiency of *Hspa8* mRNA translation in dendrites while avoiding GCN4-HSPA8-SETB accumulation (Fig. 5c and 5d). In control motor neurons, a few reporter mRNAs localized to the dendrites and were translated by monosomes or polyribosomes throughout the shaft. Likewise, somatic reporter mRNAs were translated at different efficiencies (Fig. 5c, upper panel). MG132 exposure increased the transcription and somatic and dendritic localization of reporter mRNAs (Fig. 5c). Its accumulation impaired accurate quantification of the somatic mRNA translation efficiency, although bright magenta spots depicting polyribosomes colocalized with the mRNA signal in control and MG132-stressed neurons (Fig. 5c, magnification in bottom left panel). In dendrites, colocalization between the peptide and mRNA signals revealed mRNAs translated by monosomes and polysomes at varying distances from the soma (Fig. 5c, magnification in bottom right panel). The percentage of mRNAs being translated per dendrite significantly increased upon MG132 exposure (Fig. 5d). Similarly, dendritic mRNA translation was more efficient in MG132-stressed neurons, as measured by the number of ribosomes reading a single mRNA (Fig. 5e). Thus, transcripts of the constitutive *Hspa8* mRNAs escaped the translational repression associated with MG132 ^105^ and instead its translation efficiency was increased in response to proteostasis demands. These results strongly suggest that combining increased mRNA localization and maintained translation efficiency during stress provides an on-demand source of dendritic HSPA8.

### The active transport of Hspa8 mRNAs by FUS ensures dendrite proteostasis

**Figure 6:**
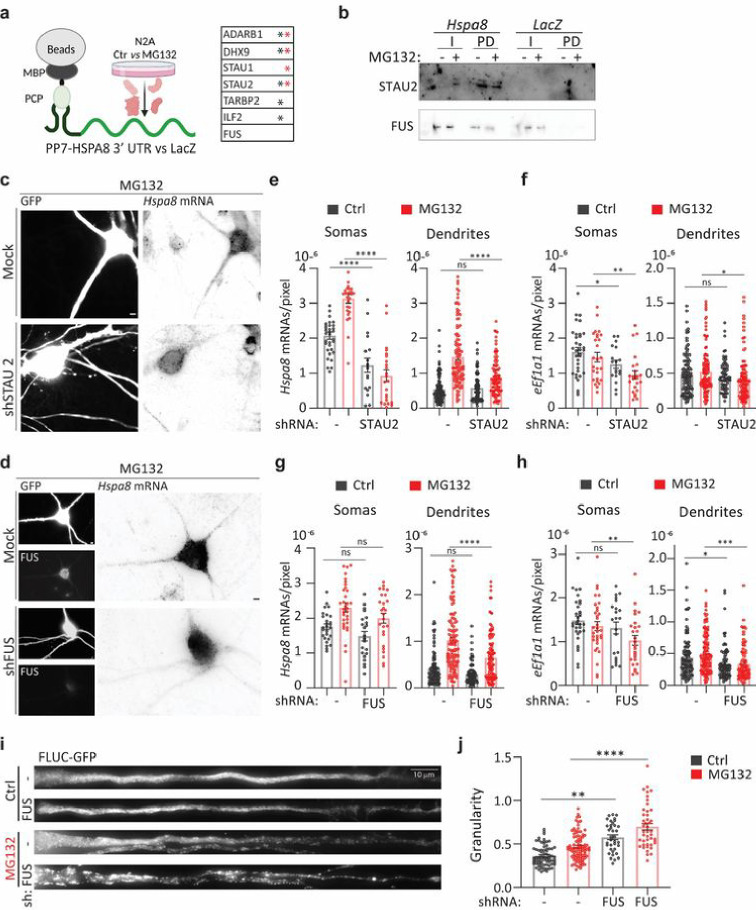
FUS regulates dendritic *Hspa8* mRNA localization and neuronal proteostasis in mouse motor neurons **(a)** Schematic of the pulldown strategy used to identify RBPs binding to the *Hspa8* 3′ UTR, and a table of the RBPs identified as specifically bound to the *HSPA8* 3′ UTR in extracts from Ctrl (black *) and MG132-stressed (red *) N2A cells by MS. **(b)** Pulldown experiments to validate the binding of STAU2 and FUS to the Hspa8 3′ UTR were analyzed by western blot. I, input; PD, pulldown. **(c, d)** Representative images of primary mouse motor neurons expressing GFP (Ctrl) or GFP and shRNAs against STAU2 (**c**) or FUS (**d**). Three days after microinjection, stress was induced with MG132, and HSPA8 mRNA expression was detected by smFISH. Scale bars = 5 mm. **(e-h)** Quantification of the densities of *Hspa8* (**d, g**) and *eEf1a1*(**f, h**) mRNAs per pixel of soma or dendrite area in Ctrl and MG132-stressed motor neurons expressing GFP with and without the indicated shRNA expression plasmids. **(i)** Representative dendrites from Ctrl and MG132-stressed motor neurons expressing the proteostasis reporter plasmid FLUC-GFP with and without FUS knockdown. GFP aggregation is proportional to proteostasis loss. **(j)** Quantification of the GFP signal granularity (the coefficient of variation) in each dendrite in I. Two independent experiments were performed (*n* = 44–96 dendrites).

Neurons contain many RBPs relevant to dendritic RNA transport, and several, including TAR DNA binding protein/TARDBP, FUS, and fragile X messenger ribonucleoprotein 1, tightly couple mRNA transport to translation, which is vital for neuronal function ^41–43^. Accordingly, mutations in these RBPs lead to diverse neurodegenerative disorders ^106^. We next identified RBPs that can mediate the dendritic transport of *Hspa8* mRNA by binding to linear or structural zip codes in its 3′ UTR (Fig. 6a). We *in vitro* transcribed the *Hspa8* 3′ UTR and LacZ (as a negative control) sequences fused to 2×PP7 stem loops and attached them to amylose magnetic beads using the PP7 capsid protein fused to the maltose binding protein (PCP-MBP) ^85^. Mass spectrometry (MS) identified six RBPs specifically bound to the *Hspa8* 3′ UTR in crude protein extracts obtained from control or MG132-stressed mouse Neuro-2A (N2A) cells (Fig. 6a). Among them, we validated the binding of staufen double-stranded RNA binding protein 2 (STAU2) because of its well-known function in stabilizing and transporting specific mRNAs to dendrites ^31,107,108^ (Fig. 6b). We also validated the binding of FUS to the *Hspa8* 3′ UTR. Although FUS was consistently present in the MS profiles of both the *Hspa8* 3′ UTR and the LacZ control, it showed relatively higher binding to the 3′ UTR in pulldowns (Fig. 6b). FUS was of particular interest because it regulates several steps in mRNA maturation, including transport to dendrites, and *FUS* mutations lead to dendritic retraction in motor neurons, leading to ALS and frontotemporal dementia ^27,109–114^.

To investigate the roles of STAU2 and FUS in the stress-induced dendritic localization of *Hspa8* mRNA, we knocked down their expression in cultured motor neurons by co-microinjecting two specific short hairpin (sh)RNAs for each along with a green fluorescent protein (GFP)-expressing plasmid to identify the injected neurons (Fig. 6c–6h **and Fig. S5a**). Knocking down STAU2 significantly decreased the somatic and dendritic density of *Hspa8* mRNA (quantified as mRNAs per pixel of soma or dendrite area) in control and MG132-stressed neurons (Fig. 6e); however, MG132 still significantly increased the density of *Hspa8* mRNA in dendrites (*p* < 0.001 by unpaired *t*-test), demonstrating that STAU2 knockdown did not prevent *Hspa8* mRNA dendritic transport. To assess specificity, we analyzed the constitutive non-HSP *eEf1a1* mRNA in parallel. STAU2 knockdown had a milder effect on *eEf1a1* mRNA density in the soma and dendrites (Fig. 6f). On the contrary, knocking down FUS did not change the somatic concentration of *Hspa8* mRNA but significantly decreased its dendritic density upon MG132 exposure (Fig. 6d and 6g), and led to an overall decrease in *eEf1a1* mRNA density in the soma and dendrites (Fig. 5h). The ratio of dendritic to somatic *eEf1a1* mRNA density did not change upon stress, whereas the FUS depleted neurons displayed decreases in these ratios (**Fig. S5b**). Thus, FUS plays an essential role in targeting Hspa8 mRNAs to dendrites during stress.

We next evaluated whether FUS knockdown could weaken dendritic proteostasis using the proteostasis reporter FLUC-GFP ^115^. Impaired proteostasis leads to GFP aggregation, which was quantified by the granularity of the GFP signal. We injected the FLUC-GFP plasmid into motor neurons either alone or with the FUS shRNA plasmids. Knocking down FUS increased GFP granularity in dendrites even under control conditions, suggesting that normal FUS levels are essential for neuronal proteostasis under physiological conditions. The loss of dendritic proteostasis upon FUS knockdown was further increased upon MG132 treatment, leading to significant increases in GFP granularity and in the sizes of the GFP aggregates (Fig. 6i and 6j). These results reveal regulated FUS expression as a determinant of neuronal proteostasis.

### The D290V mutation in HNRNPA2B1 impairs HSPA8 mRNA localization in human-derived motor neurons

**Figure 7:**
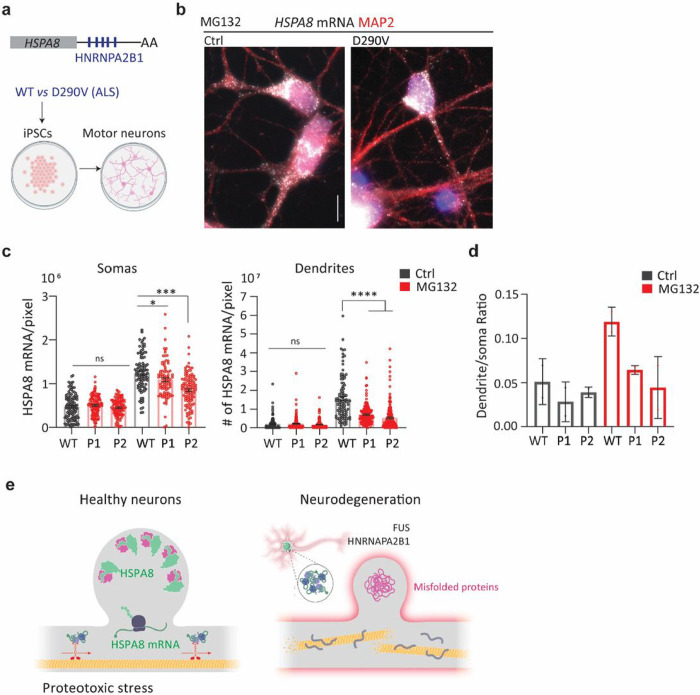
An ALS-associated *HNRAPA2B1* mutation impairs dendritic *HSPA8* mRNA localization in human motor neurons **(a)** Schematic of human HSPA8 mRNAs and the differentiation of iPSCs from healthy and D190V donors into motor neurons. **(b)** IF-smFISH to stain dendrites with an anti-MAP2 antibody and detect HSPA8 mRNAs in MG132-stressed motor neurons differentiated from healthy (WT) donors and patients with ALS carrying the HNRNAPA2B1D290V mutation. Scale bar = 10 mm. **(c)** Quantification of somatic and dendritic HSPA8 mRNAs in Ctrl and MG132-stressed human-derived motor neurons from experiment B. Data are the mean ± SEM of two independent experiments (*n* = 89–121 neurons, individual soma and dendrite values indicated by a dot). Motor neurons differentiated from healthy donors WT and patients (P). ***, *P* < 0.001; **, *P* < 0.01; *, *P* < 0.05; ns, not significant (by unpaired *t*-test). (**d**) Ratio of HSPA8 mRNA per pixel of soma or dendrite area in the MG132-stressed motor neurons analyzed in C. (**e**) Summary of conclusions.

Mutations in FUS and other RBPs, including HNRNPA2B1, lead to ALS ^116^. Through a recent collaboration, we found that mouse motor neurons microinjected with a plasmid expressing the familiar FUS^R521G^ mutation had significant lower *Hspa8* mRNAs levels in soma and dendrites (*Fernandez, M. et al, submitted to Cell Stress and Chaperones*). As the *HSPA8* 3′ UTR sequence and length differs between mice and humans, we next investigated whether human motor neurons localize HSPA8 mRNA to dendrites upon MG132 stress and which RBPs mediate their localization. Interestingly, the human *HSPA8* 3′ UTR sequence contains five putative HNRNPA2B1 binding sites that are not present in mice (Fig. 7a). In patients with ALS, the D290V mutation in HNRNPA2B1 is rare. However, it promotes the accumulation of the detergent-insoluble HNRNPA2B1 protein in the nucleus and changes the subcellular distribution of mRNAs during stress ^117^. Because of this, human motor neurons differentiated from patient fibroblast-derived induced pluripotent stem cells (iPSCs) do not recover from stress as well as neurons differentiated from healthy donors ^118,119^. Thus, we compared the ability of healthy (control) and HNRNPA2B1^D290V^-expressing motor neurons (from two patients each) to localize *HSPA8* mRNAs to the dendrites upon MG132 exposure (Fig. 7a and 7b.

Importantly, human motor neurons behaved like mouse neurons, increasing the *HSPA8* mRNA level and its dendritic localization upon MG132 stress (Fig. 7b and 7c). The D290V mutation impaired the somatic accumulation of *HSPA8* mRNAs in HNRNPA2B1^D290V^-derived motor neurons, and remarkably, both sets of HNRNPA2B1^D290V^-expressing motor neurons had significantly less dendritic *HSPA8* mRNA than healthy motor neurons and this decrease was more significant for patient 2 than patient 1 (Fig. 7c). As a result, the distribution ratio of *HSPA8* mRNA in the dendrites relative to the soma was lower in HNRNPA2B1^D290V^ motor neurons than in controls upon MG132 stress (Fig. 7d). Therefore, HNRNPA2B1’s role in localizing *HSPA8* mRNAs to dendrites is compromised by the D290V mutation, contributing to impaired proteostasis and proteotoxic damage.

Overall, decreased FUS expression and *HNRNPA2B1* mutation have similar consequences in stressed motor neurons: impaired *Hspa8* mRNA dendritic localization and decreased stress recovery. Based on our results, we propose a model in which healthy neurons sustain dendritic proteostasis through the regulated localization and translation of HSPs, especially the constitutive chaperone HSPA8, providing an on-demand system to tailor the amounts of HSPs to the load of misfolded proteins. Disruptions in RBPs that impair this localization decrease neuronal proteostatic capacity and prevent synapse formation and transmission, leading to neurodegeneration (Fig. 7e).

## Discussion

This study uncovered a novel mechanism to sustain neuronal proteostasis under proteotoxic stress; the partitioning of distinct HSPs in the soma and dendrites through the regulated localization of their encoding mRNAs and subsequent translation. Stress-induced changes in HSP mRNA compartmentalization indicate that the soma and dendrite’s distinct proteomes are upheld by particular sets of chaperones. It also suggests that after proteotoxic damage the dendrite demands for HSPs exceed the neuron capacity to transport individual chaperones from the soma and, instead rely on the competence of an mRNA to produce tens of proteins. The mRNA encoding the constitutive HSP70, HSPA8 has the highest levels of all HSP mRNAs we detected in dendrites. HSPA8 is central to sustaining proteostasis because of its chaperonin functions in assisting co-translational folding and regulated degradation of proteins by chaperone-mediated autophagy ^120^. HSPA8 also plays moonlighting functions in axonal terminals, where mediates synaptic vesicle fusion and recycling ^64,121^, and in dendrites, where it regulates the shape of dendritic spines ^122^. Besides these critical roles, how HSPA8 localizes to neuronal projections was understudy. Leveraging single mRNA imaging techniques in stressed mouse hippocampal and motor neurons in culture, has allowed us to discover a specific and regulated *Hspa8* mRNA targeting mechanism to dendrites. Since we did not detect HSP mRNAs in axons, they might localize HSPA8 synthesized in the soma.

This study identified the post-transcriptional regulation of constitutive *Hspa8* mRNA in neurons, both its boosted dendritic localization and translational efficiency, as a crucial aspect of their stress response. It operates by RBPs recognizing zip codes in the mRNAs, dynein leading them to dendrites where they are transported as individual molecules, and a stress-induced translational efficiency. As such, it resembles the induced postsynaptic localization and local translation of the mRNAs encoding an activity-regulated cytoskeleton-associated protein ARC and b-ACTIN that occurs upon synaptic stimulation ^12,25^. We approached uncovering the components directing *Hspa8* mRNAs to dendrites by searching for RBPs that bind to the 3′ UTR sequence of *Hspa8* mRNA since this region is known for its role in mRNA localization. We identified STAU2, well-known for its function in stabilizing and transporting specific mRNAs to dendrites ^31,107,108^. Although STAU2 knockdown in cultured motor neurons reduced the levels of *Hspa8* mRNAs in the soma and dendrites, it failed to prevent the increase in dendritic *Hspa8* mRNAs in response to treatment with MG132. Conversely, FUS was identified as an important player in the subcellular distribution of *Hspa8* mRNA. FUS knockdown significantly impaired *Hspa8* mRNA localization in dendrites but did not completely abrogate it. Thus, FUS might cooperate with additional RBPs binding *Hspa8* 5’ UTR or CDS. It is also possible that the observed effect of FUS to be indirect and operate through the expression regulation of factors involved in the transport of mRNAs.

Besides the fundamental role of FUS in *Hspa8* mRNA transport to dendrites upon stress, we explored this RBP because it is implicated in ALS ^116^, in which dendritic attrition is an early sign of motor neuron damage ^6,123,124^. FUS depletion correlated with a decreased *Hsap8* mRNA dendritic localization and the loss of dendritic proteostasis. Since findings in mouse neurons might not reflect the human situation because of different nucleotide sequences in the HSPA8 3′ UTR, we examined iPSC-derived motor neurons. We focused on patient-derived neurons carrying the ALS-linked HNRNPA2B1 mutation D290V because the 3’UTR of the human *HSPA8* mRNA, but not the mouse, has five putative binding sites for HNRNPA2B1. Both lines of HNRNPA2B1^D290V^-derived motor neurons had significantly less dendritic *HSPA8* mRNA than control-derived neurons, indicating that the general principles of RBP regulation of dendritic HSP mRNAs are conserved between mouse and human neurons, with common roles in the neuronal stress response. In addition, the converging actions of different disease forms on *HSPA8* mRNA biogenesis could explain the common loss of proteostasis that characterizes diverse neurodegenerative diseases.

Neurons last the lifespan of an organism, and loss of neuron proteostasis is a feature of many aging-related neurodegenerative diseases. The localized synthesis of HSPs provides dendrites with the folding resources to aid their protein synthesis and degradation mechanisms in sustaining proteostasis. Perturbations in any of the proteostasis components preclude dendritic homeostasis and lead to neurodegeneration. As such, boosting HSP transcription has been considered as a therapeutic strategy for neurodegenerative diseases, but has had limited clinical success ^125–128^, and has focused particularly on HSPA1A, which is not upregulated in neurons under most conditions ^72,125,126^. Our work stresses the importance of regulating not only the levels of constitutive HSPs, but also the dynamics of their localization to vulnerable regions, such as dendritic spines. These dynamics are crucial to consider when testing potential therapeutics. Sustaining functional synapses is essential for neuronal network functions, and stress on their proteomes would contribute to the impaired connectivity that underlies loss of function early in neurodegenerative disorders, prior to neuronal death ^129–131^.

## Methods

### Neuronal cultures

Mouse primary hippocampal neurons were obtained from postnatal day 0 C57BL/6 and FVB mice and prepared as previously described ^78,85^. Housing and euthanasia were performed in compliance with the Canadian Council on Animal Care. Neurons used in imaging experiments were cultured at low density (50,000 neurons per 35 mm (14 mm glass) dish (MatTek, # P35G-1.5–14-C)). Neurons used in RNA-seq and Simple Western experiments were cultured at 300,000 neurons per well in 100 μm Transwell membranes (Thermo Fisher Scientific, # 35102) in 6-well dishes ^80^.

Dissociated spinal cord cultures from E13 CD1 mice were prepared as previously described ^96^. Cells were plated on poly-D-lysine (Sigma, #P6407) - and Matrigel-coated glass coverslips in 6-well dishes. The culture medium was as described ^96^ with the addition of 1% B27 (Gibco Life Technologies, Burlington, ON, Canada, #17504044), 0.032 g/mL sodium bicarbonate and 0.1 g/mL dextrose. Cultures were maintained for at least 3 weeks to ensure motor neuron maturation.

Human motor neurons were differentiated from iPSCs as previously described ^117,118^. CV-B (wild type) iPSCs were a gift from the Zhang Lab ^132^ and HNRNPA2B1 D290V-1.1 and − 1.2 human iPSCs were generated in the Yeo lab ^118^. Human iPSCs were grown on Matrigel-coated 10 cm tissue culture plates. When cells were 80–90% confluent, they were split into 6-well plates at 1×10^6^ cells/well in 1× N2B27 medium (DMEM/F12 + Glutamax, 1:200 N2 supplement, 1:100 B27 supplement, 150 mM ascorbic acid, and 1% Penicillin/Streptomycin) supplemented with 1 μM dorsomorphin (Tocris, #3093), 10 μM SB431542 (Tocris, #1614), 4 μM CHIR99021 (Tocris, #4423) and 10 μM Y-27632 hydrochloride (ROCK inhibitor; Tocris, #1254). The seeding day was counted as day 1. On days 1–5, the cells were refed daily with the same medium as on day 1, but with the ROCK inhibitor reduced to 5 μM. On days 7–17, the cells were refed daily with 1× N2B27 medium supplemented with 1 μM dorsomorphin, 10 μM SB431542, 1.5 μM retinoic acid (Sigma, #R2625), 200 nM Smoothened Agonist, SAG (EMD Millipore, #566660), and 5 μM ROCK inhibitor. On day 18, the cells were either plated on laminin-coated 10 cm plates at 1.2×10^7^ cells per plate for continued differentiation or expanded in motor neuron progenitor MNP medium (1× N2B27 medium supplemented with 3 mM CHIR99021, 2 mM DMH1 (Tocris, #4126), 2 mM SB431542, 0.1 mM retinoic acid, 0.5 mM purmorphamine (Tocris, #4551), and 0.5 mM valproic acid (Tocris, #2815)) on Matrigel-coated plates. To expand motor neuron progenitors, cells were refed every other day with MNP medium. Laminin plates were prepared by serially coating them with 0.001% (0.01 mg/mL) poly-D-lysine (Sigma, #P6407) and poly-L-ornithine (Sigma, #P3655) followed by 20 μg/mL laminin (Life Technologies, #23017015). Cells were refed on day 18 and day 20 with MN medium (1 × N2B27 medium supplemented with 2 ng/mL glial cell-derived neurotrophic factor, 2 ng/mL bone-derived neurotrophic factor, and 2 ng/mL ciliary neurotrophic factor (all from R&D Systems, #212-GD, #248-BD, and #257-CF, respectively) supplemented with 1.5 μM retinoic acid, 200 nM SAG, and either 10 μM ROCK inhibitor on day 18 or 2 μM ROCK inhibitor on day 20. On days 22 and 24, cells were fed with MN medium supplemented with 2 μM DAPT and 2 μM ROCK inhibitor. On day 25, cells were split onto laminin-coated glass coverslips in a 12-well plate at 6.7×10^6^ cells/ well in MN medium supplemented with 10 μM ROCK inhibitor. On day 27, cells were fed with MN medium supplemented with 2 μM ROCK inhibitor. On day 29, cells were stressed with 10 μM MG132 (Sigma, # M7449) for 7 h at 37°C. Cells were then fixed in 4% paraformaldehyde in phosphate-buffered saline and5 mM MgCl2 (PBSM) for 1 h at room temperature, washed once with 0.1 M glycine in PBSM for 10 min, and stored in PBSM at 4°C for IF staining and mRNA FISH.

### Neuronal manipulation

Neurons were stressed via 10 μM MG132 for the indicated times, hypoxia-reoxygenation (1% O_2_ for 3 h and 4 h recovery at 5% O_2_) using a hypoxia glove box (BioSpherix Xvivo System Model X3), or incubation with oligomers made from 500 nM amyloid-β (1–42) monomers (rPeptide, #1163–1) ^133^. As mature neurons cannot be transfected, plasmids were introduced into primary cultured mouse motor neurons by intranuclear microinjection. The injectate (the plasmid in 50% Tris-ethylenediaminetetraacetic acid (EDTA), pH 7.2) was clarified by centrifugation prior to insertion into 1 mm diameter quick-fill glass capillaries (World Precision Instruments) pulled to fine tips using a Narishige PC-10 puller (Narishige International USA, Inc., NY, USA). Cultures on coverslips were bathed in Eagle’s minimum essential medium without bicarbonate, supplemented with 5 g/L glucose, and adjusted to pH 7.4 in 35 mm culture dishes on the stage of a Zeiss Axiovert 35 microscope (Carl Zeiss Microscopy, LLC, USA) and microinjected using a Transjector 5246 or a FemtoJet Transjector and a Micromanipulator 5171 (all from Eppendorf, Hamburg, Germany). Following microinjection, coverslips were placed in regular culture medium containing 0.75% Gentamicin (Gibco) and maintained at 37°C in a 5% CO_2_ environment until analysis.

### Plasmid transfection and analysis

Plasmids expressing shRNAs in a lentiviral backbone were obtained through the McGill University library (https://www.sidonghuanglab.com/pooled-screening-libraries/service-request/) (**Table S2)**. They were transfected by calcium phosphate into 293T cells and knockdown efficiency was tested 72 h later by western blotting (**Table S2**).

### IF and smFISH

Detailed protocols for these methods have been previously described ^78^. RNA FISH probes were designed using the Stellaris Probe Designer (LGC Biosearch Technologies; masking level: 5, oligo length: 20, minimum spacing: 2) (**Table S2**).

### Image acquisition and analysis

Images were acquired using a Nikon eclipse Ti-2 inverted widefield microscope equipped with a SPECTRA X Light Engine (Lumencor) and an Orca-Fusion Digital CMOS Camera (Hamamatsu) controlled by NIS-Elements Imaging Software. A 60× 1.40 NA oil immersion Plan Apochromat objective lens (Nikon) was used with a xy pixel size of 107.5 nm and a z-step of 200 nm. Chromatic aberrations were measured before imaging using 100 nm TetraSpeck^™^ Fluorescent Microspheres (Invitrogen, #T14792) and considered in the downstream pipeline.

Single mRNAs, peptides, and postsynaptic densities were identified with the MATLAB version of the FISH-quant (v3) ^86^. Post-detection analyses of subcellular mRNA distributions in neurons and simulations were performed with the second version of ARLIN ^88^. The code for ARLIN v1.0 and ARLIN v2.0 can be found here: https://github.com/LR-MVU/neuron. See the corresponding documentation for a detailed explanation on ARLIN’s functionalities. Briefly, in ARLIN v2.0, simulations were improved by mimicking the distributions of real mRNAs when selecting simulated mRNAs. To do this, the dendrite was divided into “bins” of 25 μm. The program first counts *x* real mRNAs found in the first bin of the dendrite (*i.e*., 0–25 μm from the soma). Then, the program selects *x* “simulated mRNAs” (*i.e*., randomly selected pixels) from only the first bin of the dendrite. This ensures that the concentration of simulated mRNAs near the soma matches the concentration of real mRNAs, but with random distributions within the bin. The program counts the number of real mRNAs found in each bin, then randomly selects that number of pixels within it as “simulated mRNAs”. With this improved simulation, the statistical likelihood of an mRNA localizing to the synapses or to another mRNA can be calculated. statistics are calculated for the localization of mRNAs to synapses or to another mRNA. The simulation is repeated 100 times and the localization statistics are averaged. This provides a more accurate comparison between random and biologically driven colocalization patterns than in the first version of ARLIN.

To quantify translation efficiency, cell and dendrite segmentations were performed manually using the “Define outlines” tool in FISH-quant. The smFISH and peptide spots in the cells were fit to a 3D Gaussian model based on the point spread function and the analysis was run in batch mode. The x, y, and z coordinates of the mRNAs and peptides in cells were exported as tabulated text files (.txt) recording the identity of each cell in each image file analyzed. We designed a Python pipeline to first calculate the number of nascent peptides in spots with a signal brighter to that corresponding to the the average intensity of one peptide. Secondly, the pipeline assigns each peptide to the closest mRNA in the cell. If the distance between an mRNA and its closest peptide exceeded the threshold (200 nm plus the chromatic aberration), then we considered it a non-translating mRNA. To remove repeated mRNAs and peptides within the threshold, we selected the brightest (*i.e*., brightest) peptide signal, and then the closest to a single mRNA. The percentage of translating mRNA was calculated by dividing the number of translating mRNAs by the total number of mRNAs per cell (https://github.com/LR-MVU/neuron).

We used Fiji (ImageJ 2.14.0, Java 1.9.0_322) to calculate the granularity of the FLUC-GFP signal. First, we generated a maximum projection for the GFP channel. Then, we outlined a region of interest to define each dendrite in a neuron and measured its area, mean fluorescent signal, and standard deviation (SD). The coefficient of signal variation—the SD divided by the mean—was used as a readout for GFP.

### RNA extraction and RNA-seq

After 17 days in culture^134^, primary hippocampal neurons were washed with PBS, and the somas were scraped from the membrane and placed into a tube. Somas were centrifuged for 2 min at 2,000 × *g* and resuspended in 400 μL ice-cold PBS. The somas were divided into two tubes, and 750 μL of Zymo RNA Lysis Buffer (ZymoResearch, # R1013) was added to each. To harvest the neurites, membranes were cut from the Transwell, placed face down in a 6 cm plate containing 750 μL of Zymo RNA Lysis Buffer, and incubated for 15 min on ice while tilting the plate every few minutes. The solution was transferred into an Eppendorf tube. RNA was isolated using a Zyma Quick RNA Miniprep Kit (ZymoResearch, #R1054). RNA library generation and Illumina sequencing was performed by the University of Montreal Genomic Platform. PolyA capture, Nextseq High Output paired-end run (2 × 75 bp, coverage ~ 50M paired-ends per sample). The raw data have been deposited in the Gene Expression Omnibus under accession number GSE202202.

RNA-seq analysis was performed on usegalaxy.org. Adaptors and reads with a quality below 20 within 4-base sliding windows were removed using Trimmomatic (galaxy version 0.38.0; https://doi.org/10.1093/bioinformatics/btu170). Trimmed single-end reads were aligned to the mouse mm10 genome using STAR (galaxy version 2.7.8a + galaxy0; https://doi.org/10.1093/bioinformatics/bts635) with default parameters, and the number of reads per transcript was determined using featureCounts (galaxy version 2.0.1 + galaxy2; https://doi.org/10.1093/bioinformatics/btt656) using default parameters. Differential gene expression was determined using DESeq2 (galaxy version 2.11.40.7 + galaxy1; https://doi.org/10.1186/s13059-014-0550-8) using default parameters. Gene ontology analysis to identify biological processes enriched in differentially expressed genes was performed using geneontology.org.

To validate the data by RT-qPCR, 25 ng of RNA isolated from the soma or neurites was reverse transcribed into cDNA using iScript^™^ Reverse Transcription Supermix (Bio-Rad) following the manufacturer’s instructions. For qPCR, cDNAs were diluted two-fold in water. PCR was performed in 5 μL reactions consisting of 1 μL DNA, 2.5 μL PowerUp SYBR Green Master Mix (Thermo Fisher Scientific) and 0.25 μL of each primer (at 1 μM) on a Viaa 7 Real-Time PCR System (Thermo Fisher Scientific; 45 cycles). Standard curves were generated using a log titration of N2A genomic DNA (50–0.05 ng) and used to quantify the cDNA. The primers used are listed below.

### Simple Westerns

Primary hippocampal neurons grown on Transwell membranes were treated with 10 μM MG132 for 7 h at 37°C. After washing with ice-cold PBS, somas were first scrapped from the upper part of the membrane and then neurites were scraped from the bottom membrane and resuspended in ice-cold PBS. After centrifugation, somas and neurites were resuspended in radioimmunoprecipitation buffer (150 mM NaCl, 10 mM Tris-HCl pH 7.5, 0.1% sodium dodecyl sulfate (SDS), 1% Triton X-100, 1% sodium deoxycholate, and 5 mM EDTA pH 8.0). Protein extracts from somas and neurites were stored at −80°C. Simple Westerns (Bio-Techne) were performed in multiplex on a Jess automated western blot system (Bio-Techne) following the manufacturer’s instructions (**Table S2**).

### Identification of RBPs binding to the mouse HSPA8 3′ UTR

PP7-HSPA8 and PP7-LacZ RNA were first PCR amplified from N2A genomic DNA or a plasmid (donated by Dr. Jerry Pelletier) using the primers listed below, and then *in vitro* transcribed with a MEGAshortscript^™^ T7 Transcription Kit (Invitrogen, # AM1354) following the manufacturer’s instructions. After transcription, RNA was treated with 2 units of Turbo DNase and then purified by phenol-chloroform extraction and ethanol precipitation. RNA was resuspended in 10 mM Tris containing 0.2 U/mL RNaseOUT. Small samples were resolved on a 0.5X TBE agarose gel and their A260/A280 ratios were measured using a nanodrop to verify the purity of the RNAs. PP7-HSPA8 and PP7-LacZ RNA were heated for 2 min at 95°C, allowed to cool to room temperature to allow PP7 loops to form, and stored at −80°C.

To prepare crude N2A extracts, cells were differentiated into the neuronal phenotype for 3 days: one day in DMEM supplemented with 5% fetal bovine serum (FBS) and 20 μM retinoic acid, one day in 2.5% FBS and 20 μM retinoic acid, and one day in 1.25% FBS and 20 μM retinoic acid. Half of the cells were treated with 10 μM MG132 for 7 h at 37°C. Cells were washed once with ice-cold 1× PBS and pelleted by centrifugation. The cell pellets were washed once with 1× PBS and 1 mM phenylmethylsulfonyl fluoride. The supernatant was removed and cells were stored at −80°C. The pellets were thawed on ice, resuspended in three volumes of N2A lysis buffer (50 mM Tris-HCl pH 7.5, 100 mM NaCl, 1 mM MgCl_2_, 0.1 mM CaCl_2_, 1% IGEPAL CA-360, 0.5% deoxycholic acid, 0.1% SDS, 1 mM phenylmethylsulfonyl fluoride, 1 mM dithiothreitol, 1× Complete Protease Inhibitor (Roche), and 100 U/mL RNaseOUT) and incubated on ice for 10 min. Cells were snap frozen in liquid nitrogen and thawed on ice twice before 10 min centrifugation at max speed. The crude extract (supernatant) was transferred to new tubes and stored at −80°C. Protein concentration was determined by Bradford assay and a small sample of crude extract was run on SDS-PAGE stained with Coomassie Blue to ensure no protein degradation.

In 100 μL reaction, 1.5 μM of PP7-HSPA8 3′ UTR or PP7-LacZ RNA were incubated with 2 μM MBP-PP7 in RNA-IP buffer (20 mM Tris pH 7.2, 200 mM NaCl, 1 mM EDTA pH 8.0, 5 mM dithiothreitol, and 0.01% IGEPAL CA-360) for 1 h on ice. Magnetic amylose beads (100 μL) were washed twice with RNA-IP buffer, then rotated with PP7-HSPA8 3′ UTR or PP7-LacZ bound to MBP-PP7 for 1 h at 4°C. The beads were washed twice with RNA-IP buffer then resuspended in 5 mL RNA-IP buffer supplemented with 0.01 mg/mL tRNA (Sigma, #10109541001) and 5–10 mg N2A crude extract for MS or 2 mg of crude extract for western blots. After rotating the beads and N2A crude extract for 2 h at 4°C, the beads were washed five times with RNA-IP buffer, resuspended in 50 μL RNA-IP buffer and 6 μg of TEV protease, and rotated for 3 h at 4°C. The cleaved PP7 proteins bound to the HSPA8 3′ UTR or LacZ RNA and their interactors was collected and the beads were incubated in fresh RNA-IP buffer containing TEV protease overnight. The elutions were pooled, and the proteins were analyzed by MS as previously described (Proteomics RIMUHC-McGill University)^135^. Proteins with fold change values > 1.5 and P-values < 0.01 compared to in the control sample were considered HSPA8 3′ UTR interactors. Statistics performed using total spectral count and a T-test analysis.

## References

[1] Costa-Mattioli M., Sossin W. S., Klann E. & Sonenberg N. Translational Control of Long-Lasting Synaptic Plasticity and Memory. Neuron 61, 10–26 (2009). 19146809 10.1016/j.neuron.2008.10.055 PMC5154738

[2] Costa-Mattioli M. Translational control of hippocampal synaptic plasticity and memory by the eIF2α kinase GCN2. Nature 436, 1166–1170 (2005). 16121183 10.1038/nature03897 PMC1464117

[3] Huber K. M., Kayser M. S. & Bear M. F. Role for Rapid Dendritic Protein Synthesis in Hippocampal mGluR-Dependent Long-Term Depression. Science 288, 1254–1256 (2000). 10818003 10.1126/science.288.5469.1254

[4] Kang H. & Schuman E. M. A Requirement for Local Protein Synthesis in Neurotrophin-Induced Hippocampal Synaptic Plasticity. Science 273, 1402–1406 (1996). 8703078 10.1126/science.273.5280.1402

[5] Miller S. Disruption of Dendritic Translation of CaMKIIα Impairs Stabilization of Synaptic Plasticity and Memory Consolidation. Neuron 36, 507–519 (2002). 12408852 10.1016/s0896-6273(02)00978-9

[6] Nakano I. & Hirano A. Atrophic Cell Processes of Large Motor Neurons in the Anterior Horn in Amyotrophic Lateral Sclerosis: Observation with Silver Impregnation Method. J Neuropathol Exp Neurol 46, 40–49 (1987). 2432193 10.1097/00005072-198701000-00004

[7] Hoffman P. M., Pitts O. M., Bilello J. A. & Cimino E. F. Retrovirus induced motor neuron degeneration. Rev Neurol (Paris) 144, 676–679 (1988). 2852840

[8] Poulopoulos A. Subcellular transcriptomes and proteomes of developing axon projections in the cerebral cortex. Nature 565, 356–360 (2019). 30626971 10.1038/s41586-018-0847-y PMC6484835

[9] Cagnetta R., Frese C. K., Shigeoka T., Krijgsveld J. & Holt C. E. Rapid Cue-Specific Remodeling of the Nascent Axonal Proteome. Neuron 99, 29–46.e4 (2018). 30008298 10.1016/j.neuron.2018.06.004 PMC6048689

[10] Holt C. E., Martin K. C. & Schuman E. M. Local translation in neurons: visualization and function. Nat Struct Mol Biol 26, 557–566 (2019). 31270476 10.1038/s41594-019-0263-5

[11] Glock C. The mRNA translation landscape in the synaptic neuropil. 10.1101/2020.06.09.141960 (2020) doi:10.1101/2020.06.09.141960.

[12] Yoon Y. J. Glutamate-induced RNA localization and translation in neurons. Proc Natl Acad Sci USA 113, E6877–E6886 (2016). 27791158 10.1073/pnas.1614267113 PMC5098659

[13] Donlin-Asp P. G., Polisseni C., Klimek R., Heckel A. & Schuman E. M. Differential regulation of local mRNA dynamics and translation following long-term potentiation and depression. Proc. Natl. Acad. Sci. U.S.A. 118, e2017578118 (2021). 33771924 10.1073/pnas.2017578118 PMC8020670

[14] Raghuraman R., Benoy A. & Sajikumar S. Protein Synthesis and Synapse Specificity in Functional Plasticity. in The Oxford Handbook of Neuronal Protein Synthesis (ed. Sossin W. S.) 268–296 (Oxford University Press, 2021). doi:10.1093/oxfordhb/9780190686307.013.16.

[15] Steward O., Wallace C. S., Lyford G. L. & Worley P. F. Synaptic Activation Causes the mRNA for the IEG Arc to Localize Selectively near Activated Postsynaptic Sites on Dendrites. Neuron 21, 741–751 (1998). 9808461 10.1016/s0896-6273(00)80591-7

[16] Steward O. & Levy W. Preferential localization of polyribosomes under the base of dendritic spines in granule cells of the dentate gyrus. J. Neurosci. 2, 284–291 (1982). 7062109 10.1523/JNEUROSCI.02-03-00284.1982 PMC6564334

[17] Kulkarni V. V. Synaptic activity controls autophagic vacuole motility and function in dendrites. Journal of Cell Biology 220, e202002084 (2021). 33783472 10.1083/jcb.202002084 PMC8020715

[18] Bingol B. & Schuman E. M. Activity-dependent dynamics and sequestration of proteasomes in dendritic spines. Nature 441, 1144–1148 (2006). 16810255 10.1038/nature04769

[19] Sun C. An abundance of free regulatory (19 S) proteasome particles regulates neuronal synapses. Science 380, eadf2018 (2023). 37228199 10.1126/science.adf2018

[20] Ramachandran K. V. & Margolis S. S. A mammalian nervous-system-specific plasma membrane proteasome complex that modulates neuronal function. Nat Struct Mol Biol 24, 419–430 (2017). 28287632 10.1038/nsmb.3389 PMC5383508

[21] Torre E. & Steward O. Demonstration of local protein synthesis within dendrites using a new cell culture system that permits the isolation of living axons and dendrites from their cell bodies. J. Neurosci. 12, 762–772 (1992). 1545238 10.1523/JNEUROSCI.12-03-00762.1992 PMC6576031

[22] Rao A. & Steward O. Evidence that protein constituents of postsynaptic membrane specializations are locally synthesized: analysis of proteins synthesized within synaptosomes. J. Neurosci. 11, 2881–2895 (1991). 1880554 10.1523/JNEUROSCI.11-09-02881.1991 PMC6575261

[23] Loedige I. mRNA stability and m6A are major determinants of subcellular mRNA localization in neurons. Molecular Cell 83, 2709–2725.e10 (2023). 37451262 10.1016/j.molcel.2023.06.021 PMC10529935

[24] Das S., Singer R. H. & Yoon Y. J. The travels of mRNAs in neurons: do they know where they are going? Current Opinion in Neurobiology 57, 110–116 (2019). 30784978 10.1016/j.conb.2019.01.016 PMC6650148

[25] Das S., Lituma P. J., Castillo P. E. & Singer R. H. Maintenance of a short-lived protein required for long-term memory involves cycles of transcription and local translation. Neuron 111, 2051–2064.e6 (2023). 37100055 10.1016/j.neuron.2023.04.005 PMC10330212

[26] Sun C. The prevalence and specificity of local protein synthesis during neuronal synaptic plasticity. Sci. Adv. 7, eabj0790 (2021). 34533986 10.1126/sciadv.abj0790 PMC8448450

[27] Fernandopulle M. S., Lippincott-Schwartz J. & Ward M. E. RNA transport and local translation in neurodevelopmental and neurodegenerative disease. Nat Neurosci (2021) doi:10.1038/s41593-020-00785-2. PMC8860725 33510479

[28] Das S., Vera M., Gandin V., Singer R. H. & Tutucci E. Intracellular mRNA transport and localized translation. Nat Rev Mol Cell Biol 22, 483–504 (2021). 33837370 10.1038/s41580-021-00356-8 PMC9346928

[29] Ravanidis S., Kattan F.-G. & Doxakis E. Unraveling the Pathways to Neuronal Homeostasis and Disease: Mechanistic Insights into the Role of RNA-Binding Proteins and Associated Factors. IJMS 19, 2280 (2018). 30081499 10.3390/ijms19082280 PMC6121432

[30] Roegiers F. Insights into mRNA transport in neurons. Proc. Natl. Acad. Sci. U.S.A. 100, 1465–1466 (2003). 12578967 10.1073/pnas.0630376100 PMC149851

[31] Doyle M. & Kiebler M. A. Mechanisms of dendritic mRNA transport and its role in synaptic tagging: Mechanisms of dendritic mRNA transport. The EMBO Journal 30, 3540–3552 (2011). 21878995 10.1038/emboj.2011.278 PMC3181491

[32] Kobayashi H., Yamamoto S., Maruo T. & Murakami F. Identification of a *cis* -acting element required for dendritic targeting of activity-regulated cytoskeleton-associated protein mRNA. European Journal of Neuroscience 22, 2977–2984 (2005). 16367764 10.1111/j.1460-9568.2005.04508.x

[33] Rodrigues E. C., Grawenhoff J., Baumann S. J., Lorenzon N. & Maurer S. P. Mammalian Neuronal mRNA Transport Complexes: The Few Knowns and the Many Unknowns. Front. Integr. Neurosci. 15, 692948 (2021). 34211375 10.3389/fnint.2021.692948 PMC8239176

[34] Hirokawa N., Niwa S. & Tanaka Y. Molecular Motors in Neurons: Transport Mechanisms and Roles in Brain Function, Development, and Disease. Neuron 68, 610–638 (2010). 21092854 10.1016/j.neuron.2010.09.039

[35] Dienstbier M., Boehl F., Li X. & Bullock S. L. Egalitarian is a selective RNA-binding protein linking mRNA localization signals to the dynein motor. Genes Dev. 23, 1546–1558 (2009). 19515976 10.1101/gad.531009 PMC2704466

[36] Bullock S. L., Nicol A., Gross S. P. & Zicha D. Guidance of Bidirectional Motor Complexes by mRNA Cargoes through Control of Dynein Number and Activity. Current Biology 16, 1447–1452 (2006). 16860745 10.1016/j.cub.2006.05.055

[37] Kiebler M. A. & Bassell G. J. Neuronal RNA Granules: Movers and Makers. Neuron 51, 685–690 (2006). 16982415 10.1016/j.neuron.2006.08.021

[38] Sossin W. S. & DesGroseillers L. Intracellular Trafficking of RNA in Neurons. Traffic 7, 1581–1589 (2006). 17054760 10.1111/j.1600-0854.2006.00500.x

[39] Thelen M. P. & Kye M. J. The Role of RNA Binding Proteins for Local mRNA Translation: Implications in Neurological Disorders. Front. Mol. Biosci. 6, 161 (2020). 32010708 10.3389/fmolb.2019.00161 PMC6974540

[40] Liao Y.-C. RNA Granules Hitchhike on Lysosomes for Long-Distance Transport, Using Annexin A11 as a Molecular Tether. Cell 179, 147–164.e20 (2019). 31539493 10.1016/j.cell.2019.08.050 PMC6890474

[41] Chu J.-F., Majumder P., Chatterjee B., Huang S.-L. & Shen C.-K. J. TDP-43 Regulates Coupled Dendritic mRNA Transport-Translation Processes in Co-operation with FMRP and Staufen1. Cell Reports 29, 3118–3133.e6 (2019). 31801077 10.1016/j.celrep.2019.10.061

[42] Yasuda K. The RNA-binding protein Fus directs translation of localized mRNAs in APC-RNP granules. Journal of Cell Biology 203, 737–746 (2013). 24297750 10.1083/jcb.201306058 PMC3857475

[43] Urbanska A. S. ZBP1 phosphorylation at serine 181 regulates its dendritic transport and the development of dendritic trees of hippocampal neurons. Sci Rep 7, 1876 (2017). 28500298 10.1038/s41598-017-01963-2 PMC5431813

[44] Young J. C., Agashe V. R., Siegers K. & Hartl F. U. Pathways of chaperone-mediated protein folding in the cytosol. Nat Rev Mol Cell Biol 5, 781–791 (2004). 15459659 10.1038/nrm1492

[45] Jayaraj G. G., Hipp M. S. & Hartl F. U. Functional Modules of the Proteostasis Network. Cold Spring Harb Perspect Biol 12, a033951 (2020). 30833457 10.1101/cshperspect.a033951 PMC6942124

[46] Sala A. J., Bott L. C. & Morimoto R. I. Shaping proteostasis at the cellular, tissue, and organismal level. J. Cell Biol. 216, 1231–1241 (2017). 28400444 10.1083/jcb.201612111 PMC5412572

[47] Alagar Boopathy L. R., Jacob-Tomas S., Alecki C. & Vera M. Mechanisms tailoring the expression of heat shock proteins to proteostasis challenges. Journal of Biological Chemistry 101796 (2022) doi:10.1016/j.jbc.2022.101796. 35248532 PMC9065632

[48] Wentink A. S. Molecular dissection of amyloid disaggregation by human HSP70. Nature 587, 483–488 (2020). 33177717 10.1038/s41586-020-2904-6

[49] Kampinga H. H. Guidelines for the nomenclature of the human heat shock proteins. Cell Stress and Chaperones 14, 105–111 (2009). 18663603 10.1007/s12192-008-0068-7 PMC2673902

[50] Campanella C. Heat Shock Proteins in Alzheimer’s Disease: Role and Targeting. IJMS 19, 2603 (2018). 30200516 10.3390/ijms19092603 PMC6163571

[51] Rosenzweig R., Nillegoda N. B., Mayer M. P. & Bukau B. The Hsp70 chaperone network. Nat Rev Mol Cell Biol 20, 665–680 (2019). 31253954 10.1038/s41580-019-0133-3

[52] Abisambra J. F. Phosphorylation Dynamics Regulate Hsp27-Mediated Rescue of Neuronal Plasticity Deficits in Tau Transgenic Mice. Journal of Neuroscience 30, 15374–15382 (2010). 21084594 10.1523/JNEUROSCI.3155-10.2010 PMC3073547

[53] Gamerdinger M. Protein quality control during aging involves recruitment of the macroautophagy pathway by BAG3. EMBO J 28, 889–901 (2009). 19229298 10.1038/emboj.2009.29 PMC2647772

[54] Liu Q., Liang C. & Zhou L. Structural and functional analysis of the Hsp70/Hsp40 chaperone system. Protein Science 29, 378–390 (2020). 31509306 10.1002/pro.3725 PMC6954727

[55] Petrucelli L. CHIP and Hsp70 regulate tau ubiquitination, degradation and aggregation. Human Molecular Genetics 13, 703–714 (2004). 14962978 10.1093/hmg/ddh083

[56] Kumar P. CHIP and HSPs interact with β-APP in a proteasome-dependent manner and influence Aβ metabolism. Human Molecular Genetics 16, 848–864 (2007). 17317785 10.1093/hmg/ddm030

[57] Carra S., Seguin S. J., Lambert H. & Landry J. HspB8 Chaperone Activity toward Poly(Q)-containing Proteins Depends on Its Association with Bag3, a Stimulator of Macroautophagy. J. Biol. Chem. 283, 1437–1444 (2008). 18006506 10.1074/jbc.M706304200

[58] Eroglu B., Moskophidis D. & Mivechi N. F. Loss of Hsp110 Leads to Age-Dependent Tau Hyperphosphorylation and Early Accumulation of Insoluble Amyloid β. MCB 30, 4626–4643 (2010). 20679486 10.1128/MCB.01493-09 PMC2950521

[59] Chaari A. Molecular chaperones biochemistry and role in neurodegenerative diseases. International Journal of Biological Macromolecules 131, 396–411 (2019). 30853582 10.1016/j.ijbiomac.2019.02.148

[60] Lindquist S. The Heat-Shock Response. Annu. Rev. Biochem. 55, 1151–1191 (1986). 2427013 10.1146/annurev.bi.55.070186.005443

[61] Wolff S., Weissman J. S. & Dillin A. Differential Scales of Protein Quality Control. Cell 157, 52–64 (2014). 24679526 10.1016/j.cell.2014.03.007

[62] Parsell D. A. & Lindquist S. The Function of Heat-Shock Proteins in Stress Tolerance: Degradation and Reactivation of Damaged Proteins. Annu. Rev. Genet. 27, 437–496 (1993). 8122909 10.1146/annurev.ge.27.120193.002253

[63] Suzuki T. Presence of molecular chaperones, heat shock cognate (Hsc) 70 and heat shock proteins (Hsp) 40, in the postsynaptic structures of rat brain. Brain Research 816, 99–110 (1999). 9878698 10.1016/s0006-8993(98)01083-x

[64] Coyne A. N. Post-transcriptional Inhibition of Hsc70–4/HSPA8 Expression Leads to Synaptic Vesicle Cycling Defects in Multiple Models of ALS. Cell Reports 21, 110–125 (2017). 28978466 10.1016/j.celrep.2017.09.028 PMC5679478

[65] Kim J.-K. A spinal muscular atrophy modifier implicates the SMN protein in SNARE complex assembly at neuromuscular synapses. Neuron 111, 1423–1439.e4 (2023). 36863345 10.1016/j.neuron.2023.02.004 PMC10164130

[66] Gorenberg E. L. & Chandra S. S. The Role of Co-chaperones in Synaptic Proteostasis and Neurodegenerative Disease. Front. Neurosci. 11, 248 (2017). 28579939 10.3389/fnins.2017.00248 PMC5437171

[67] Brown I. R. Heat Shock Proteins at the Synapse: Implications for Functional Protection of the Nervous System. in Heat Shock Proteins and the Brain: Implications for Neurodegenerative Diseases and Neuroprotection (eds. Asea A. A. A. & Brown I. R.) 239–254 (Springer Netherlands, 2008). doi:10.1007/978-1-4020-8231-3_12.

[68] Bechtold D. A. & Brown I. R. Heat shock proteins Hsp27 and Hsp32 localize to synaptic sites in the rat cerebellum following hyperthermia. Molecular Brain Research 75, 309–320 (2000). 10686353 10.1016/s0169-328x(99)00323-x

[69] Bechtold D. A., Rush S. J. & Brown I. R. Localization of the Heat-Shock Protein Hsp70 to the Synapse Following Hyperthermic Stress in the Brain. Journal of Neurochemistry 74, 641–646 (2001). 10.1046/j.1471-4159.2000.740641.x 10646515

[70] Gomez-Pastor R., Burchfiel E. T. & Thiele D. J. Regulation of heat shock transcription factors and their roles in physiology and disease. Nat Rev Mol Cell Biol 19, 4–19 (2018). 28852220 10.1038/nrm.2017.73 PMC5794010

[71] Anckar J. & Sistonen L. Regulation of HSF1 function in the heat stress response: implications in aging and disease. Annu. Rev. Biochem. 80, 1089–1115 (2011). 21417720 10.1146/annurev-biochem-060809-095203

[72] Vera M. The translation elongation factor eEF1A1 couples transcription to translation during heat shock response. eLife 3, e03164 (2014). 25233275 10.7554/eLife.03164 PMC4164936

[73] Vera M. & Singer R. H. Gene regulation: the HSP70 gene jumps when shocked. Curr. Biol. 24, R396–398 (2014). 24845669 10.1016/j.cub.2014.03.070 PMC4156153

[74] Middleton S. A., Eberwine J. & Kim J. Comprehensive catalog of dendritically localized mRNA isoforms from sub-cellular sequencing of single mouse neurons. BMC Biol 17, 5 (2019). 30678683 10.1186/s12915-019-0630-z PMC6344992

[75] Das S., Lituma P. J., Castillo P. E. & Singer R. H. Cycles of transcription and local translation support molecular long-term memory in the hippocampus. 10.1101/2021.10.29.466479 (2021) doi:10.1101/2021.10.29.466479. PMC10330212 37100055

[76] Seibenhener M. L. & Wooten M. W. Isolation and Culture of Hippocampal Neurons from Prenatal Mice. JoVE 3634 (2012) doi:10.3791/3634. PMC3476399 22871921

[77] Lituma P. J., Singer R. H., Das S. & Castillo P. E. Real-time imaging of Arc/Arg3.1 transcription ex vivo reveals input-specific immediate early gene dynamics. 10.1101/2021.12.16.472958 (2021) doi:10.1101/2021.12.16.472958. PMC9499544 36095210

[78] Jacob-Tomas S., Alagar Boopathy L. R. & Vera M. Using Single-Molecule Fluorescence Microscopy to Uncover Neuronal Vulnerability to Protein Damage. in Neuronal Cell Death (ed. Jahani-Asl A.) vol. 2515 237–254 (Springer US, 2022). 10.1007/978-1-0716-2409-8_15 35776356

[79] Türker F., Cook E. K. & Margolis S. S. The proteasome and its role in the nervous system. Cell Chemical Biology 28, 903–917 (2021). 33905676 10.1016/j.chembiol.2021.04.003 PMC8286317

[80] Poon M. M., Choi S.-H., Jamieson C. A. M., Geschwind D. H. & Martin K. C. Identification of Process-Localized mRNAs from Cultured Rodent Hippocampal Neurons. Journal of Neuroscience 26, 13390–13399 (2006). 17182790 10.1523/JNEUROSCI.3432-06.2006 PMC6675000

[81] Love M. I., Huber W. & Anders S. Moderated estimation of fold change and dispersion for RNA-seq data with DESeq2. Genome Biol 15, 550 (2014). 25516281 10.1186/s13059-014-0550-8 PMC4302049

[82] Brehme M. A Chaperome Subnetwork Safeguards Proteostasis in Aging and Neurodegenerative Disease. Cell Reports 9, 1135–1150 (2014). 25437566 10.1016/j.celrep.2014.09.042 PMC4255334

[83] Gerges N. Z. Independent Functions of hsp90 in Neurotransmitter Release and in the Continuous Synaptic Cycling of AMPA Receptors. Journal of Neuroscience 24, 4758–4766 (2004). 15152036 10.1523/JNEUROSCI.0594-04.2004 PMC6729466

[84] Femino A. M. Visualization of Single RNA Transcripts in Situ. Science 280, 585–590 (1998). 9554849 10.1126/science.280.5363.585

[85] Eliscovich C., Shenoy S. M. & Singer R. H. Imaging mRNA and protein interactions within neurons. Proc Natl Acad Sci USA 114, E1875–E1884 (2017). 28223507 10.1073/pnas.1621440114 PMC5347572

[86] Mueller F. FISH-quant: automatic counting of transcripts in 3D FISH images. Nat. Methods 10, 277–278 (2013). 23538861 10.1038/nmeth.2406

[87] Imbert A. FISH-quant v2: a scalable and modular tool for smFISH image analysis. RNA rna.079073.121 (2022) doi:10.1261/rna.079073.121. PMC9074904 35347070

[88] Wefers Z., Alecki C., Huang R., Jacob-Tomas S. & Vera M. Analysis of the Expression and Subcellular Distribution of eEF1A1 and eEF1A2 mRNAs during Neurodevelopment. Cells 11, 1877 (2022). 35741005 10.3390/cells11121877 PMC9220863

[89] Schneider J. L. & Cuervo A. M. Chaperone-mediated autophagy: dedicated saviour and unfortunate victim in the neurodegeneration arena. Biochemical Society Transactions 41, 1483–1488 (2013). 10.1042/BST20130126 PMC4000258 24256241

[90] Lackie R. E. The Hsp70/Hsp90 Chaperone Machinery in Neurodegenerative Diseases. Front. Neurosci. 11, 254 (2017). 28559789 10.3389/fnins.2017.00254 PMC5433227

[91] Chen R. Reactive Oxygen Species Formation in the Brain at Different Oxygen Levels: The Role of Hypoxia Inducible Factors. Front. Cell Dev. Biol. 6, 132 (2018). 30364203 10.3389/fcell.2018.00132 PMC6192379

[92] Lee, Reggie H. C. Cerebral ischemia and neuroregeneration. Neural Regen Res 13, 373 (2018). 29623912 10.4103/1673-5374.228711 PMC5900490

[93] Yeo E.-J. Hypoxia and aging. Exp Mol Med 51, 1–15 (2019). 10.1038/s12276-019-0233-3 PMC6586788 31221957

[94] Resenberger U. K. The Heat Shock Response Is Modulated by and Interferes with Toxic Effects of Scrapie Prion Protein and Amyloid β. Journal of Biological Chemistry 287, 43765–43776 (2012). 23115236 10.1074/jbc.M112.389007 PMC3527961

[95] Sackmann C. & Hallbeck M. Oligomeric amyloid-β induces early and widespread changes to the proteome in human iPSC-derived neurons. Sci Rep 10, 6538 (2020). 32300132 10.1038/s41598-020-63398-6 PMC7162932

[96] Roy J., Minotti S., Dong L., Figlewicz D. A. & Durham H. D. Glutamate Potentiates the Toxicity of Mutant Cu/Zn-Superoxide Dismutase in Motor Neurons by Postsynaptic Calcium-Dependent Mechanisms. J. Neurosci. 18, 9673–9684 (1998). 9822728 10.1523/JNEUROSCI.18-23-09673.1998 PMC6793286

[97] Baas P. W. & Lin S. Hooks and comets: The story of microtubule polarity orientation in the neuron. Developmental Neurobiology 71, 403–418 (2011). 21557497 10.1002/dneu.20818 PMC3151545

[98] Quintyne N. J. Dynactin Is Required for Microtubule Anchoring at Centrosomes. The Journal of Cell Biology 147, 321–334 (1999). 10525538 10.1083/jcb.147.2.321 PMC2174233

[99] Krichevsky A. M. & Kosik K. S. Neuronal RNA Granules. Neuron 32, 683–696 (2001). 11719208 10.1016/s0896-6273(01)00508-6

[100] Batish M., Van Den Bogaard P., Kramer F. R. & Tyagi S. Neuronal mRNAs travel singly into dendrites. Proc. Natl. Acad. Sci. U.S.A. 109, 4645–4650 (2012). 22392993 10.1073/pnas.1111226109 PMC3311338

[101] Wu B., Eliscovich C., Yoon Y. J. & Singer R. H. Translation dynamics of single mRNAs in live cells and neurons. Science 352, 1430–1435 (2016). 27313041 10.1126/science.aaf1084 PMC4939616

[102] Yan X., Hoek T. A., Vale R. D. & Tanenbaum M. E. Dynamics of Translation of Single mRNA Molecules In Vivo. Cell 165, 976–989 (2016). 27153498 10.1016/j.cell.2016.04.034 PMC4889334

[103] Morisaki T. Real-time quantification of single RNA translation dynamics in living cells. Science 352, 1425–1429 (2016). 27313040 10.1126/science.aaf0899

[104] Wang C., Han B., Zhou R. & Zhuang X. Real-Time Imaging of Translation on Single mRNA Transcripts in Live Cells. Cell 165, 990–1001 (2016). 27153499 10.1016/j.cell.2016.04.040 PMC4905760

[105] Shelkovnikova T. A. Chronically stressed or stress-preconditioned neurons fail to maintain stress granule assembly. Cell Death Dis 8, e2788–e2788 (2017). 28492545 10.1038/cddis.2017.199 PMC5520719

[106] Gamarra M., de la Cruz A., Blanco-Urrejola M. & Baleriola J. Local Translation in Nervous System Pathologies. Front. Integr. Neurosci. 15, 689208 (2021). 34276318 10.3389/fnint.2021.689208 PMC8279726

[107] Heraud-Farlow J. E. Staufen2 Regulates Neuronal Target RNAs. Cell Reports 5, 1511–1518 (2013). 24360961 10.1016/j.celrep.2013.11.039

[108] Köhrmann M. Microtubule-dependent Recruitment of Staufen-Green Fluorescent Protein into Large RNA-containing Granules and Subsequent Dendritic Transport in Living Hippocampal Neurons. MBoC 10, 2945–2953 (1999). 10473638 10.1091/mbc.10.9.2945 PMC25535

[109] Sephton C. F. Activity-dependent FUS dysregulation disrupts synaptic homeostasis. Proc. Natl. Acad. Sci. U.S.A. 111, (2014). 10.1073/pnas.1406162111 PMC4226112 25324524

[110] Sévigny M. FUS contributes to mTOR-dependent inhibition of translation. Journal of Biological Chemistry 295, 18459–18473 (2020). 33082139 10.1074/jbc.RA120.013801 PMC7939483

[111] Shiihashi G. Dendritic Homeostasis Disruption in a Novel Frontotemporal Dementia Mouse Model Expressing Cytoplasmic Fused in Sarcoma. EBioMedicine 24, 102–115 (2017). 28928015 10.1016/j.ebiom.2017.09.005 PMC5652009

[112] Imperatore J. A., McAninch D. S., Valdez-Sinon A. N., Bassell G. J. & Mihailescu M. R. FUS Recognizes G Quadruplex Structures Within Neuronal mRNAs. Front. Mol. Biosci. 7, 6 (2020). 32118033 10.3389/fmolb.2020.00006 PMC7018707

[113] Ishigaki S. & Sobue G. Importance of Functional Loss of FUS in FTLD/ALS. Front. Mol. Biosci. 5, 44 (2018). 29774215 10.3389/fmolb.2018.00044 PMC5943504

[114] Fujii R. The RNA Binding Protein TLS Is Translocated to Dendritic Spines by mGluR5 Activation and Regulates Spine Morphology. Current Biology 15, 587–593 (2005). 15797031 10.1016/j.cub.2005.01.058

[115] Blumenstock S. Fluc-EGFP reporter mice reveal differential alterations of neuronal proteostasis in aging and disease. EMBO J 40, (2021). 10.15252/embj.2020107260 PMC8488555 34410010

[116] Butti Z. & Patten S. A. RNA Dysregulation in Amyotrophic Lateral Sclerosis. Front. Genet. 9, 712 (2019). 30723494 10.3389/fgene.2018.00712 PMC6349704

[117] Martinez F. J. Protein-RNA Networks Regulated by Normal and ALS-Associated Mutant HNRNPA2B1 in the Nervous System. Neuron 92, 780–795 (2016). 27773581 10.1016/j.neuron.2016.09.050 PMC5123850

[118] Markmiller S. Persistent mRNA localization defects and cell death in ALS neurons caused by transient cellular stress. Cell Reports 36, 109685 (2021). 34496257 10.1016/j.celrep.2021.109685 PMC11341010

[119] Krach F. Aberrant NOVA1 function disrupts alternative splicing in early stages of amyotrophic lateral sclerosis. Acta Neuropathol 144, 413–435 (2022). 35778567 10.1007/s00401-022-02450-3 PMC9381448

[120] Cuervo A. M. & Dice J. F. Age-related Decline in Chaperone-mediated Autophagy. Journal of Biological Chemistry 275, 31505–31513 (2000). 10806201 10.1074/jbc.M002102200

[121] Blatnik A. J. & Macleod Burghes A. H. An Hspa8 variant is a shocking modifier of spinal muscular atrophy in mice. Neuron 111, 1349–1350 (2023). 37141858 10.1016/j.neuron.2023.03.025

[122] Yagi H., Takabayashi T., Xie M.-J., Kuroda K. & Sato M. Subcellular distribution of non-muscle myosin IIb is controlled by FILIP through Hsc70. PLoS ONE 12, e0172257 (2017). 28234934 10.1371/journal.pone.0172257 PMC5325215

[123] Kabashi E. Gain and loss of function of ALS-related mutations of TARDBP (TDP-43) cause motor deficits in vivo. Human Molecular Genetics 19, 671–683 (2010). 19959528 10.1093/hmg/ddp534

[124] Tradewell M. L. Arginine methylation by PRMT1 regulates nuclear-cytoplasmic localization and toxicity of FUS/TLS harbouring ALS-linked mutations. Human Molecular Genetics 21, 136–149 (2012). 21965298 10.1093/hmg/ddr448

[125] Batulan Z. High threshold for induction of the stress response in motor neurons is associated with failure to activate HSF1. J. Neurosci. 23, 5789–5798 (2003). 12843283 10.1523/JNEUROSCI.23-13-05789.2003 PMC6741252

[126] Kuta R. Depending on the stress, histone deacetylase inhibitors act as heat shock protein co-inducers in motor neurons and potentiate arimoclomol, exerting neuroprotection through multiple mechanisms in ALS models. Cell Stress and Chaperones 25, 173–191 (2020). 31900865 10.1007/s12192-019-01064-1 PMC6985055

[127] Kieran D. Treatment with arimoclomol, a coinducer of heat shock proteins, delays disease progression in ALS mice. Nat Med 10, 402–405 (2004). 15034571 10.1038/nm1021

[128] Elliott E. Therapeutic Targeting of Proteostasis in Amyotrophic Lateral Sclerosis—a Systematic Review and Meta-Analysis of Preclinical Research. Front. Neurosci. 14, 511 (2020). 32523508 10.3389/fnins.2020.00511 PMC7261930

[129] Campisi J. From discoveries in ageing research to therapeutics for healthy ageing. Nature 571, 183–192 (2019). 31292558 10.1038/s41586-019-1365-2 PMC7205183

[130] Selkoe D. J. Alzheimer’s Disease Is a Synaptic Failure. Science 298, 789–791 (2002). 12399581 10.1126/science.1074069

[131] Smith D. L., Pozueta J., Gong B., Arancio O. & Shelanski M. Reversal of long-term dendritic spine alterations in Alzheimer disease models. Proc. Natl. Acad. Sci. U.S.A. 106, 16877–16882 (2009). 19805389 10.1073/pnas.0908706106 PMC2743726

[132] Gore A. Somatic coding mutations in human induced pluripotent stem cells. Nature 471, 63–67 (2011). 21368825 10.1038/nature09805 PMC3074107

[133] Stine W. B., Dahlgren K. N., Krafft G. A. & LaDu M. J. In Vitro Characterization of Conditions for Amyloid-β Peptide Oligomerization and Fibrillogenesis. Journal of Biological Chemistry 278, 11612–11622 (2003). 12499373 10.1074/jbc.M210207200

[134] Arora A., Goering R., Lo H.-Y. G. & Taliaferro J. M. Mechanical Fractionation of Cultured Neuronal Cells into Cell Body and Neurite Fractions. Bio-protocol 11, e4048–e4048 (2021). 34250214 10.21769/BioProtoc.4048 PMC8250387

[135] Alagar Boopathy L. R. The ribosome quality control factor Asc1 determines the fate of HSP70 mRNA on and off the ribosome. Nucleic Acids Research gkad338 (2023) doi:10.1093/nar/gkad338. PMC10325905 37158240

